# Expression Analysis of *TCP* Transcription Factor Family in Autopolyploids of *Chrysanthemum nankingense*

**DOI:** 10.3389/fpls.2022.860956

**Published:** 2022-06-02

**Authors:** Zhongyu Yu, Chang Tian, Yunxiao Guan, Jun He, Zhenxing Wang, Likai Wang, Sisi Lin, Zhiyong Guan, Weimin Fang, Sumei Chen, Fei Zhang, Jiafu Jiang, Fadi Chen, Haibin Wang

**Affiliations:** State Key Laboratory of Crop Genetics and Germplasm Enhancement, Key Laboratory of Landscaping, Key Laboratory of Biology of Ornamental Plants in East China, Ministry of Agriculture and Rural Affairs, National Forestry and Grassland Administration, College of Horticulture, Nanjing Agricultural University, Nanjing, China

**Keywords:** polyploids, TCP transcription factors, transcriptional regulation, GA pathway, gene expression changes

## Abstract

Autopolyploids often exhibit plant characteristics different from their diploid ancestors and are frequently associated with altered genes expression controlling growth and development. TCP is a unique transcription factor family in plants that is closely related to plant growth and development. Based on transcriptome sequencing of *Chrysanthemum nankingense*, 23 full-length *TCP* genes were cloned. The expression of *CnTCP9* was most variable in tetraploids, at least threefold greater than diploids. Due to the lack of a *C. nankingense* transgenic system, we overexpressed *CnTCP9* in *Arabidopsis thaliana* (Col-0) and *Chrysanthemum morifolium*. Overexpression of *CnTCP9* caused enlargement of leaves in *A. thaliana* and petals in *C. morifolium*, and the expression of genes downstream of the GA pathway in *C. morifolium* were increased. Our results suggest that autopolyploidization of *C. nankingense* led to differential expression of TCP family genes, thereby affecting plant characteristics by the GA pathway. This study improves the understanding of enlarged plant size after autopolyploidization.

## Introduction

Polyploids formed by the same species through spontaneous genome duplication or artificial doubling of the chromosomes are designated as autopolyploids. For example, potato is a naturally formed autotetraploid whereas the autopolyploids of barley, tobacco, and rape are induced through chemical methods (Liu and Sun, 2017; Marfil et al., 2018; Yang et al., 2019). Polyploidization often causes genome instability, chromosome imbalances, regulatory incompatibilities, and reproductive failures, making the polyploid face the challenge of coordinating distinct sub-genomes with independent genetics and epigenetics from a single nucleus (Comai, 2005). Rapid increases in gene and genome dosages in autopolyploids and allopolyploids often cause genome instability, and surging genetic and epigenetic alterations occur under genomic shock (Chen, 2007; Wang et al., 2013a,^2014^; Bird et al., 2018). In polyploids, the regulation of transcription is altered by gene duplication, which provides additional flexibility to adapt and evolve new patterns of gene expression (Li et al., 2012; Wang et al., 2015; Ramirez-Gonzalez et al., 2018). Generally, increases in ploidy can result in obvious morphological changes, which is one of the most common characteristics of polyploidy (Sattler et al., 2016). Compared to their parents, most polyploids show enlarged cell volume, which often results in a limited enlargement of plant leaves, petals, stems, organs, and tissues (Liu et al., 2011; Li et al., 2014; del Pozo and Ramirez-Parra, 2015). Widespread polyploidy suggests that these unstable states always enabled polyploids to adapt to different environments owing to their increased diversity and plasticity (Jiao et al., 2011). After polyploidization, one of the sub-genomes may become dominant over the others leading to higher homologous gene expression; this dominance may represent a signature of the evolutionary process (Thomas et al., 2006; Chang et al., 2010). Many gene families play important roles in plant growth and development. Whole-genome duplication (WGD) contributes to the expansion of gene families that have likely played very important roles during evolution with some of them showing diverse expression patterns after polyploidization (Nardeli et al., 2018; Zhao et al., 2018, 2019; Kuang et al., 2020). Current research on different gene families is not extensive. The study of many key gene families between different ploidy levels of the same material helps to systematically study its functional characteristics or potential evolutionary direction.

The TCP family of transcription factors is named after the TEOSINTE BRANCHED1 (TB1) in maize, CYCLOIDEA (CYC) in goldfish grass, and PROLIFERATING CELL FACTORS 1 and 2 (PCF1 and PCF2) in rice. The TCP domain is composed of 59 amino acids with an atypical bHLH (basic-helixI-loop-helixII) structure. The TCP domains are differentially conserved in each part, with the basic past being the most conserved. The basic region consists of approximately 21 amino acids and contains a nuclear localization signal (NLS). The helix regions are less conservative, the helixI region consists of conserved hydrophilic and hydrophobic residues that form an amphipathic structure. The helixII region contains the LXXLL-motif and three phosphorylation sites. The loop region is the least conservative. The different TCP domains, can be grouped into two classes: Class I (PCF as representative) and Class II (represented by CYC and TB1) which are further categorized into two subgroups, CIN and CYC/TB1 (Cubas et al., 1999; Li, 2015).

TCP transcription factors are known to regulate plant growth and morphogenesis by interacting with other proteins or binding to other gene promoters (Li et al., 2019; Zhang et al., 2019b). Studies have shown that Class I and Class II genes in TCP family have opposite functions. Class I transcription factors promote plant growth and cell proliferation, whereas class II transcription factors inhibit growth (Zheng et al., 2018). *PCF* is a representative class I gene. In rice, the *Proliferating Cell Nuclear Antigen* (*PCNA*) gene is only expressed in proliferating cells, which is related to cell cycle progression (Kosugi and Ohashi, 1997). PCF1 and PCF2 proteins have been reported to bind to the *PCNA* gene promoter, thus promoting cell proliferation and organ growth in rice (Kosugi and Ohashi, 1997). In the auxin signaling pathway, *AtTCP15* can promote expression of the downstream genes *IAA3*/*SHY2* and *SAUR65*, which may interact with CIN-like proteins (Uberti-Manassero et al., 2012). Overexpression of *AtTCP20* caused a low germination rate, which suggests that it could play a role in cell division and expansion (Li et al., 2005; Herve et al., 2009; Steiner et al., 2012). Additionally, *AtTCP20* acts upstream of *AtTCP9* and can interact with NLP6&7 proteins controlling leaf development and root meristem growth (Danisman et al., 2012; Guan et al., 2017). Generally, TCP transcription factors can be used as mediators of hormonal activity in the process of cell proliferation, and they can also act as modulators or even key players in hormone synthesis, transport, and signal transduction in tissues and organs (Gray, 2004; Brian, 2010; Hou, 2013; Cubas and Nicolas, 2016). Previous findings showed that the Arabidopsis class I TCP transcription factors could regulate leaf development through jasmonic acid (JA) and GA metabolism (Danisman et al., 2012; Guan et al., 2017). The plant hormones GA and JA are two types of essential phytohormones that act in response to environmental and endogenous signals. GA is a class of tetracyclic diterpenoid phytohormones that regulate major aspects of plant growth and development, such as increased inter-node extension, increased leaf growth and enhanced apical dominance on shoot growth, increased dry weight, breaking seed dormancy, stem elongation, and regulated flowering (Gray, 2004; Brian, 2010). JA is a class of lipid-derived small molecules that play a central role in mediating plant responses to stress, such as regulating plant defense against herbivorous arthropods and pathogen infection, as well as plant responses to abiotic stresses, such as ozone and UV light (Gray, 2004; Hou, 2013).

The regulation of gene expression is a complex process in organisms. Studies of polyploid plants indicate that polyploidization may result in the activation or inhibition of gene expression (Chen, 2010). Owing to the great similarity of gene sequences in polyploids, the study of expression regulation between homologous genes is more complicated, especially in autopolyploids. Diploid *C. nankingense* was treated with colchicine to obtain autotetraploid plants. Compared to diploids, the tetraploids showed reduced height and number of branches, but increased stem diameter, leaf thickness, small flower size (tongue-shaped flowers and tubular flowers), and pollen grains, also deeper leaf color (Liu et al., 2011). These large differences between the diploids and tetraploids warrant a study of gene expansion and expression. The TCP family of transcription factors has been widely reported to be involved in the development of plant phenotype (Du et al., 2017; Bresso et al., 2018; Liu et al., 2018; Wang et al., 2018). In view of the significant phenotypic changes in tetraploid *C. nankingense*, we aimed to clone the TCP family, analyze its expression pattern after polyploidization. This study will further explore the effect of genome shock on gene expression patterns in chrysanthemums.

## Materials and Methods

### Plant Materials and Growth Conditions

The diploid and tetraploid of *C. nankingense* and *C. morifolium* “Jinba” were obtained from the Chrysanthemum Germplasm Resource Preserving Center, Nanjing Agricultural University (Nanjing, China). The plants were grown in a 3:1 (v/v) mixture of vermiculite and soil rite with a day/night temperature of 26/18°C and relative humidity of 70% grown in a greenhouse. The plants were grown under a 16 h light/8 h dark long day period, while *C. morifolium* plants were transferred to a 8 h light/16 h dark short day conditions after 4 weeks long day growth for inducing flowering.

### Transcriptome Sequencing, Assembly, and Annotation

Two mixed samples of aerial parts in diploid and tetraploid plants at the vegetative growth stage were used for transcriptome sequencing separately (SRA PRJNA810751). Total RNA was extracted using a Total RNA Isolation System (Takara, Japan) according to the manufacturer’s instructions. The quality of the resulting RNA was verified using Agilent 2100 (Santa Clara, CA, United States), all extractions delivered RIN > 6.3 and 28S/18S ratio > 1.5. Nanodrop ND-430 1000 spectrophotometer was used to test sample purity A260/A280 nm readings between 1.8 and 2.2, and A260/A230 nm readings greater than 2.0. Oligo dT magnetic beads were used to enrich mRNA with poly A tail, and RNase H to selectively digest DNA/RNA hybrid chain, and DNA was then digested with DNase I to obtain the required RNA after purification. The interrupted buffer was used to fragment the obtained RNA, followed by reverse transcription with random N6 primers and two-strands cDNA synthesis. Specific adapters were connected at both ends of double-stranded DNA. The ligation product is amplified by PCR with specific primers. The PCR product is thermally denatured into single-stranded, and then the single-stranded DNA is circularized with a bridge primer to obtain a single-stranded circular DNA library. After RNA extraction, purification, quality inspection, and construction of the cDNA library, Illumina Hiseq 2000 platform (Illumina, San Diego, CA, United States) was used for next-generation sequencing. Reads with low quality, contaminated linkers, and high N content of unknown bases. The filtered data is called clean reads. Trinity (Seif et al., 2021) was used for *de novo* assembly. The summary of sequencing and assembly were shown in Supplementary Tables 1, 2. The sequence library was obtained by assembling the contigs into unigenes, and removing redundancy. The non-redundant protein database (NR), non-redundant nucleotide database (NT), Swiss-Prot, Gene Ontology (GO), and COG database (The Cluster of Orthologous Groups) were used for annotation and homologous classification. Estscan^1^ was used to predict the direction of the remaining sequences. The RPKM reads (clean reads per kilo base per million) method was used to calculate the expression level of All-Unigene, which directly used for comparing the difference of gene expression among samples.

### Cloning and Analysis of *CnTCP* Family

Transcriptomic data of various tissues from vegetative to reproductive stages of diploid *C. nankingense* were used for transcriptome data assembly, including the *C. nankingense* transcriptome data from this study and previously published articles (PRJNA244464; PRJNA219225) (Wang et al., 2013b; Ren et al., 2014; Sun et al., 2014). According to the assembly results, all 23 *TCP* gene fragments were selected for verification. After obtaining the intermediate fragment, 3′-RACE and 5′-RACE specific primers were designed for nested PCR. The 3′ and 5′ sequences were obtained and assembled, and the ORF was predicted using ORF finder,^2^ and then full-length primers were designed (Supplementary Table 3).

Using the TCP family genes of Arabidopsis and rice, together with all the *TCP* genes cloned from *C. nankingense*, MEGA 5.0^3^ was used for amino acid alignment, and a phylogenetic tree was constructed using the maximum likelihood algorithm, bootstrap 1000.

### Expression Verification of *CnTCP* Family

RNA was extracted from diploid and tetraploid *C. nankingense*, each with three biological replicates. Total RNA isolation and reverse transcription were carried out following the methods described by Wang et al. (2013b) and Ren et al. (2014). In this experiment, the total expression level was determined. A 20 μL system was used for real-time RT-PCR consisting of 5 μL cDNA (∼100 ng) in each system, 10 μL 2 × SYBR^®^ Premix Ex TaqTM II, 0.6 μL primer (10 μM), and 3.8 μL ddH_2_O. The quantitative reaction was carried out using a Mastercycler^®^ ep realplex 2 S (Eppendorf, Hamburg, Germany). The chrysanthemum *EF1*α gene (Genbank accession number KF305681) was used as the reference (Ren et al., 2014). The Ct value for each target gene was normalized to the reference gene Ct value. The relative value of gene expression was derived using the 2^–ΔΔCT^ method. Three biological replicates were performed for each sample.

### Subcellular Localization and Transcriptional Activity Analysis

The amplified *CnTCP9* sequence was inserted into *pBI121* between *Bam*HI and *Sma*I to generate the expression vector *pBI121-CnTCP9*. *Allium cepa L.* epidermis was transformed using a gene gun and cultured in a dark box for 24 h. The subcellular localization of fluorescence was observed using a Leica CP SP2 laser confocal microscope.

The *CnTCP9* gene was inserted between *Nde*I and *Bam*HI to obtain the yeast expression vector *pGBKT7*- *CnTCP9.* The pGBKT7 and pCL1 were set as negative control and positive control, respectively. Then, according to the Stratagene standard yeast transformation procedure (Clontech cat. No. 630439), the plasmids were transformed into yeast *Y2H* and pre-cultured in solid culture medium at 30^°^C (negative control and yeast carrying *CnTCP9* was incubated on SD/-Trp culture medium; the positive control pCL1 was incubated on SD/-Leu culture medium). When the colonies grew to about 2 mm, a single colony was selected and inoculated on SD/-Ade/-His nutrient deficient culture medium and X-α-gal color medium at 30°C to observe colony growth.

### Generation of Transgenic Plants

The ORF of *CnTCP9* was cloned into the *pMD19-T* simple vector, double digested with *Sal*I and *Pst*I, and ligated into the pCAMBIA1301 vector. *Arabidopsis thaliana* (Col-0) was transformed by floral dip (Clough and Bent, 1998) with *Agrobacterium* (*EHA105*) containing pCAMBIA1301*-CnTCP9*. T_1_ generation seeds were screened on a selective medium containing 10 mg L^–1^ hygromycin for 21 days. DNA was extracted from the resistant seedlings using the CTAB method. PCR was performed using primers of *CnTCP9*, and the resistance was further screened for T_3_. GUS staining of T_3_ generation seedlings was performed according to the method described by Jefferson et al. (1987).

The *CnTCP9* overexpression construct was used for chrysanthemum transformation, which was performed *via* leaf disk infection as described previously (Cui et al., 2009). Briefly, leaf disks were taken from the young leaves of 30–40 d tissue culture plantlets and cut to approximately 25 mm^2^ with wounds on all sides. Transgenic plants were selected on MS medium containing 10 mg L^–1^ hygromycin. After regeneration, RNA was extracted from the putative transgenic and WT plants, digested with RNase-free DNase I (TaKaRa, Tokyo, Japan), and reverse transcribed with Reverse Transcription M-MLV (TaKaRa). For primary screening, the vector primers of the hygromycin and 2 × pro35S sequences were used to detect positive transgenic plants. The positive transgenic plants (*OX-CnTCP9*) were analyzed *via* RT-PCR to determine the relative expression levels using the primers *CnTCP9*. The expression of GA pathway-related genes (*CPSCPS, KO1, KAO1, GA2ox1, GA3ox1*, and *GA20ox2*) was also verified in the overexpressed transgenic lines. Three biological replicates and three technical replicates were performed for each sample.

### Phenotypic Investigation of Chrysanthemum

Phenotypes of WT and *OX-CnTCP9* transgenic *C. morifolium* ‘‘Jinba’’ at 80, 87, and 94 days were observed after planting in the field. Five inflorescences were selected randomly to measure the diameters of the WT and *OX-CnTCP9* transgenic plants. The lengths of the 3 outermost whorl petals of each inflorescence were measured. 25 mm^2^ pieces were dissected from the center of the middle abaxial epidermis of each petal, and the cells were observed and photographed using an Olympus BX41 optical microscope (Olympus Optical Co., Ltd., Tokyo, Japan). For cell length and width measurements, five cells in the center of three photographs of each material were performed by using ImageJ (NIH, MD, United States).^4^ Data from the individual measurements were averaged and analyzed using SPSS Statistics v. 18.0 (SPSS Inc., Chicago, IL, United States).

### Transformation Process of *Y2H* Screening

The *CnTCP9* amplification products and pGBKT7 vectors were simultaneously digested by *Bam*HI and *Eco*RI (Takara, Dalian, China). After enzymatic digestion, the *CnTCP9* fragment was ligated to pGBKT7 for 1 h at 16°C using T4 ligase (Takara) and then transformed into *DH5*α competent *E. coli* cells, from which a positive plasmid was extracted. The *Y2H* strain was spread on solid YPDA medium and cultured at 28^°^C for 3 days. Monoclone was taken and incubated overnight at 28°C and 200 rpm in liquid YPDA for shock culture. Three mL of bacterial solution was transferred into 100 mL liquid YPDA and incubated at 28^°^C 200 rpm for shock culture until OD_600_ reached 0.4–0.5. This bacterial solution was then transferred to a 50 mL centrifuge tube and centrifuged at 2,000 rpm for 5 min at 28^°^C. The supernatant was removed and the pellet was resuspended in 30 mL sterile water. After a second centrifugation at 2,000 rpm for 5 min at 28^°^C, the supernatant was removed and 1.5 mL of 1 × TE/LiAC was added to each centrifuge tube. The solution was then transferred to 2 mL centrifuge tubes and centrifuged at a high speed (12,000 rpm) for 15 s. The supernatant was then removed and the precipitation in each 2 ml centrifuge tube was resuspended in 600 μL of 1.1 × TE/LiAC. After the cells were in the receptive state, 5 μg of pGBKT7-*CnTCP9* plasmid, 10 μg of yeast pGADT7 library and 20 μl of pre-denatured salmon sperm DNA were placed in pre-cooled sterilized 10 mL centrifuge tubes, then 600 μL of receptive cells were added and gently mixed. Three milliliter of PEG/LiAC was added and mix gently. The mixture was incubated at 28^°^C and 200 rpm for 45 min for shock culture. Next, 160 μL of DMSO was added, mixed and heat-shocked in a 42^°^C water bath for 20 min, with gentle rotation every 10 min to complete the transformation. The mixture was centrifuged at 2,000 rpm for 5 min, supernatant was discarded and pellet was resuspended in 3 mL YPDPlus and incubated at 28^°^C, 200 rpm for 90 min shock culture. The mixture was again centrifuged at 2,000 rpm for 5 min, 3 mL 1 × TE was added and 100 μL of the resuspended bacterial solution was inoculated into solid SD/-Trp/-His/-Leu/-Ade plates. The plates were incubated at 28^°^C for 3 days. The monoclone was transferred into solid SD/-Trp/-His/-Leu/-Ade + X-α-gal plates cultured at 28^°^C for 3 days. The monoclonal clones that turned blue were selected for PCR detection. 5 μL of PCR products were taken for gel electrophoresis, and single bands were selected for sequencing. The sequences obtained were eventually aligned in NCBI to predict their functions.

### Hormone Content Determination

About 0.5 g of leaf tissue from each sample was ground in mortar and pestle. One milliliter of pre-cooled 70–80% methanol solution (PH = 3.5) was added for overnight extraction at 4°C. The solution was centrifuged at 12,000 g, 4^°^C for 10 min and the residue was resuspended with 0.5 mL of 70–80% methanol solution, extracted for 2 h at 4^°^C, centrifuged and the supernatant was removed. Equal volume of petroleum ether was added after evaporation to 1/3 of original volume at 40^°^C under reduced pressure. PVPP was added, incubated with shaking for 20 min, centrifuged, and the PH of supernatant was adjusted to 3.0 with hydrochloric acid. The supernatant was extracted with ethyl acetate for 3 times, ester phases were combined and evaporated to dryness at 40°C under reduced pressure. The mobile phase solution was then added, followed by vortexing and shaking and filtered with needle filter. The samples were homogenized and extracted in 1 ml of methanol: acetic acid: water [12:3:5 (v/v/v)] for 12 h at 4°C. After centrifugation for 10 min at 8,000 g (4^°^C), the supernatant was used to determine gibberellin (GA) content, and the pellet was extracted three times. The extraction solution was concentrated with a vacuum rotary evaporator. The residue was dissolved with 0.5 ml methanol: acetic acid: water [12:3:5 (v/v/v)] and filtered through a 0.22-μm filter prepared for HPLC. The HPLC liquid phase conditions: Shimadzu LC-20AT, UV detector SPD-20A, column temperature chamber CTO-20AC, C18 reversed-phase column (150 mm×4.6 mm, 5 μm); mobile phase A: 100% methanol; B: 0.1% acetic acid aqueous solution A:B = 55:45; injection volume 20 μL, flow rate 0.8 mL/min, column temperature 30°C. UV wavelength 254 nm. There were three replicates.

## Results

### Total 23 TCP Genes Were Identified in *Chrysanthemum nankingense*

**FIGURE 1:**
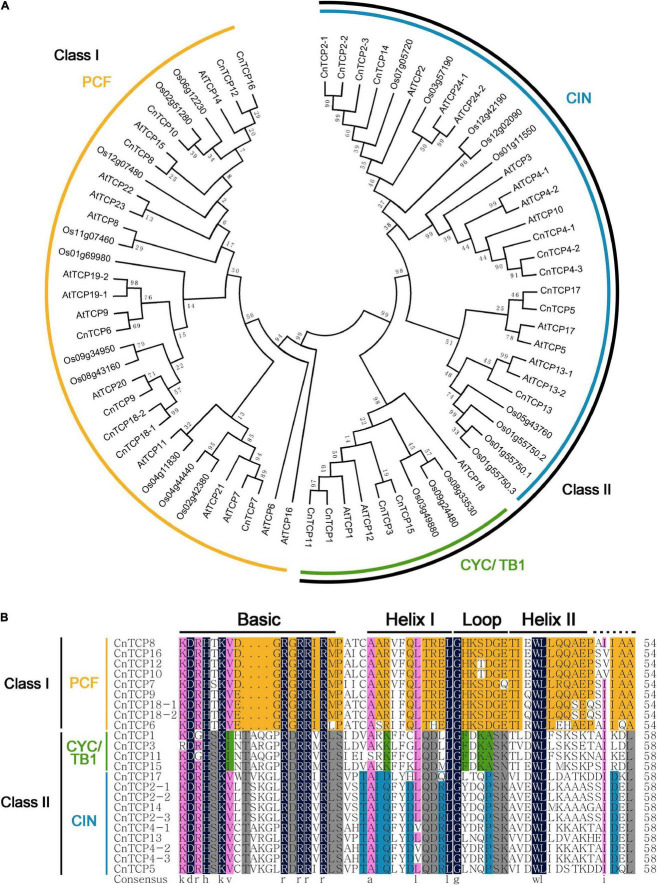
Evolutionary tree and amino acid alignment. **(A)** The phylogeny of the amino acid sequences from *C. nankingense*, rice and Arabidopsis TCP family. **(B)** Alignment of the amino acid sequences from *C. nankingense* TCP family. Amino acids are expressed in the standard single letter code. Black boxes highlight residues conserved in both TCP classes; yellow, residues conserved in class I; gray, conserved in class II; green, conserved in CYC/TB1 proteins; blue, conserved in CIN-like proteins.

Using the transcriptome data of *C. nankingense*, after splicing and removing redundancy, 21 related contigs of the *CnTCP* genes were identified. After cloning all 21 contigs using 5′-RACE and 3′-RACE, a total of 23 full-length *CnTCP* gene sequences were obtained. After sequence similarity analysis, sequences with similar lengths and consistencies (>90%) were named as different subtypes of the same gene (Genbank accession numbers 2510473 and 2510481). Finally, all *CnTCP* genes were identified and named according to their similarity to *CnTCP1*-*CnTCP18*. The highly similar sequences were *CnTCP2-1*, *2-2*, *2-3*; *CnTCP4-1, 4-2*, *4-3*; and *CnTCP18-1*, *18-2*. Among them, *CnTCP1* was the shortest (690 bp), while *CnTCP2-1* (1,335 bp) encoded the longest TCP amino acid. Sequence analysis showed that all CnTCP proteins were highly homologous with the TCPs in rice and Arabidopsis. The accession no. or locus ID of all the *TCP* genes were shown in Supplementary Table 4. The phylogenetic tree showed that CnTCP could be divided into two groups: Class I and Class II (Figure 1A). The PCF proteins of Arabidopsis and rice were all located in Class I. Class II included two branches, CYC/TB1 and CIN. The results showed that all CnTCPs aligned close to their Arabidopsis and rice orthologs and belonged to the PCF (Class I) and CYC/TB1, CIN (Class II) subfamilies, respectively.

Since all TCPs contain an atypical bHLH domain, Class I had four fewer amino acids than the Class II TCP in this conserved domain, and other regions including two helixes and one loop are relatively conserved. Multiple sequences alignment of all CnTCP domains revealed that all of them had typical basic domains, one group comprising of 17 aa (Class I) and another with 21 aa (Class II). In the other HLH structural regions, the helixI region, the rotation region and the helixII region consisted of 11, 7 and 15 conserved amino acids, respectively (Figure 1B). Based on the overall structure of bHLH in the TCP domain, 23 cloned *CnTCP* genes had atypical bHLH domains, and the classification results were consistent with the phylogenetic tree analysis.

### Expression Profiles of *CnTCP* Genes and GA and Jasmonic Acid Pathways

**FIGURE 2:**
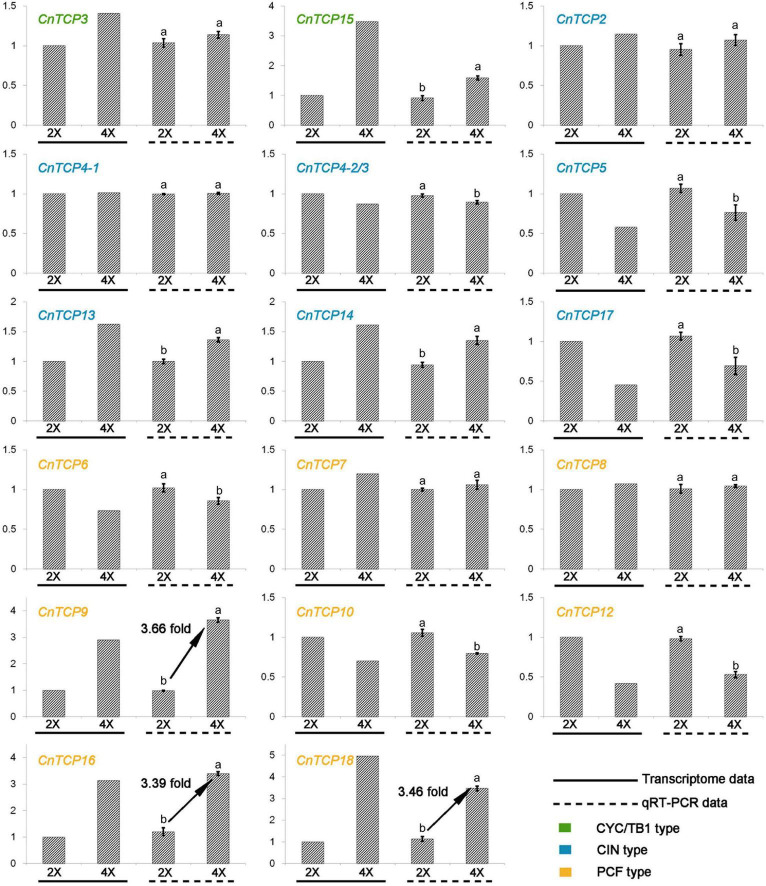
qRT-PCR verification of *CnTCP* genes. qRT-PCR analysis of *CnTCP* genes in diploid and tetraploid *C. nankingense.* The y-axes represent genes relative expression levels. Data are means ± *SD*. The different letters represent the values significantly different at *p* < 0.01 according to the *t*-test.

An analysis of gene expression patterns can provide important information about gene functions. Of all the 18 *CnTCP* genes identified and cloned from the chrysanthemum transcriptome database, 16 *CnTCP* genes were expressed in aerial parts of tetraploids and diploids *C. nankingense*, while no expression were seen for *CnTCP1* and *CnTCP11*. Since no TCP genes specifically expressed in diploid or tetraploid *C. nankingense*, we concluded that no new genes emerged or eliminated after autopolyploidization (Figure 2). The expression levels of all detected *CnTCP* genes in the diploids and tetraploids were shown in Figure 2 and Supplementary Table 6. Overall, the expression level of the CYC/TB1 subfamily was the lowest amongst all three subfamilies. The two undetected *CnTCP* genes also belonged to this subfamily. Out of a total of 11 genes with a RPKM of more than 10, 3 (*CnTCP2-1*/2/*3*) were in the CIN subfamily and 8 were in the PCF subfamily. The highest expression of 60.8 was observed in *CnTCP9*. With the increase in ploidy, 16 *CnTCP* genes were differentially expressed of which 10 were upregulated and 6 were downregulated. Among the differentially expressed genes, two belonged to the CYC/TB1 subfamily, six belonged to the CIN subfamily, and eight belonged to the PCF subfamily (Figure 2 and Supplementary Table 6). In the PCF subfamily, the higher expression changes appear on *CnTCP18-1/2*, reaching 4.95 times increased followed by *CnTCP16* (3.14 times) and *CnTCP9* (2.90 times). Overall, the existence of the largest number of differentially expressed genes and the highest average expression made the PCF subfamily the most sensitive family after polyploidization in *C. nankingense*. To verify the transcriptome data, qRT-PCR was used to quantitatively analyze the cloned *CnTCP* genes. Correlation analysis between RNA-seq and qRT-PCR data was shown in Supplementary Figure 1, which showed a strong correlation between them. The results showed that the expression trends of all the *CnTCP* genes were consistent with the results of the transcriptome analysis. In the PCF subfamily, *CnTCP9* showed the highest increase of gene expression with 3.66 fold-change in tetraploid compare to diploid *C. nankingense* followed by *CnTCP18-1/2* (3.46 times) and *CnTCP16* (3.39 times) (Figure 2).

**FIGURE 3:**
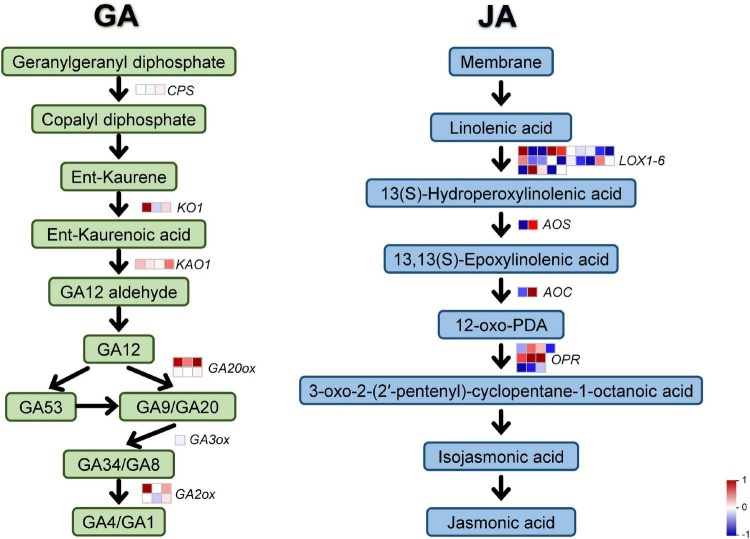
Synthesis pathway of GA and JA. The GA and JA synthesis pathway were distinguished using green and blue, rounded rectangle represented a synthesis product, arrows represented the synthesis process, squares represented the key genes in this synthesis process. The differential expression fold-change were homogenized. Red represents up regulation and blue represents down regulation.

It is known that GA signaling regulates many critical biological events in plants, like inflorescence development, leaf growth, stem elongation, while JA often play a central role in mediating plant responses to stress. To investigate whether polyploidy of *C. nankingense* affects plant characteristics through GA and JA signaling pathways, we analyzed JA and GA synthesis pathways related-genes expression patterns through transcriptomic data. In Figure 3, the two hormone synthesis pathways are represented by green and blue for GA and JA, respectively, each rounded rectangle represented a synthesis product, and the arrows represented the synthesis process, and the squares on the arrows represented the key genes in the synthesis process. Gene expression was compared using the foldchange value of tetraploidy/diploidy, if it was red, it means the gene was up-regulated in the tetraploidy compared to the diploidy, if it was blue, it means down-regulated. Meanwhile, all differentially expressed genes were homogenized. All genes and their foldchange values were listed in Supplementary Table 7. Both GA and JA pathways have many differentially expressed genes, most of the GA pathway genes were upregulated, while the number of upregulated and downregulated genes in the JA pathway was not markedly different (Figure 3 and Supplementary Table 7). The higher expression changes of GA pathway related gene appeared in *KO1* (*cl15326.contig1_all*) reaching 3.64 times more, followed by *GA2ox* (*unigene13271_all*, 1.03 times) and *GA20ox* (*unigene12251_all*, 1.27 times). In JA pathway, most of *Lipoxygenase* (*Lox*) genes, *allene oxide synthase* (*AOS*), and *12-oxophytodienoate reductase* (*OPR*) genes were significantly down-regulated, while some *Lox* genes, *OPR* genes, and *allene oxide cyclase* (*AOC*) were significantly up-regulated (Supplementary Table 7).

### *CnTCP9* Was Identified as a Key Candidate Gene Associated With Organ Enlargement in After Polypolidization

**FIGURE 4:**
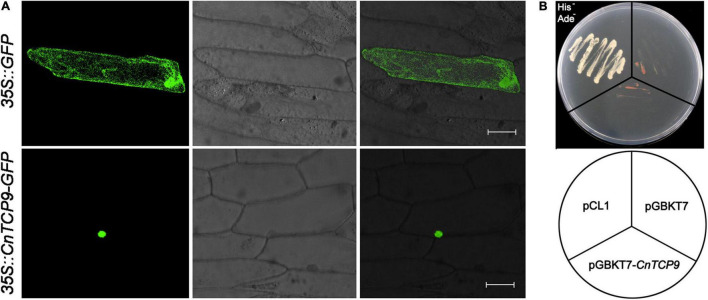
Subcellular localization and transcriptional activation. **(A)** Subcellular localization of CnTCP9 protein in the onion epidermis cells. Onion epidermis cells were transformed with 35S:GFP as a control; bar = 50 μm. **(B)** Transcriptional activation assays of CnTCP9 *via* yeast one-hybrid system.

The *CnTCP9* gene with high expression level and the most significant expression changes after polyploidization was selected for further analysis. In the subsequent functional verification, the *CnTCP9* gene was placed upstream of the *GFP* gene. The results of gene gun-mediated transformation of onion epidermal cells showed that green fluorescence was expressed in the nucleus and cell membrane of the cells transformed with 35S:*GFP* positive control, while green fluorescence was only found in the nucleus of the onion cells transformed with GFP (35S:*CnTCP9*—*GFP*) carrying CnTCP9, indicating that CnTCP9 was localized in the nucleus (Figure 4A). The results of transcriptional activation activity showed that the positive control could activate the expression of nutritional deficiency genes in yeast, whereas the full-length protein of CnTCP9 could not activate the expression of these genes. The yeast could not grow normally on SD/Ade-/His-nutritional deficiency medium, suggesting that CnTCP9 did not have transcriptional activation activity (Figure 4B). Therefore, it appears that *CnTCP9* plays a regulatory role in other genes.

**FIGURE 5:**
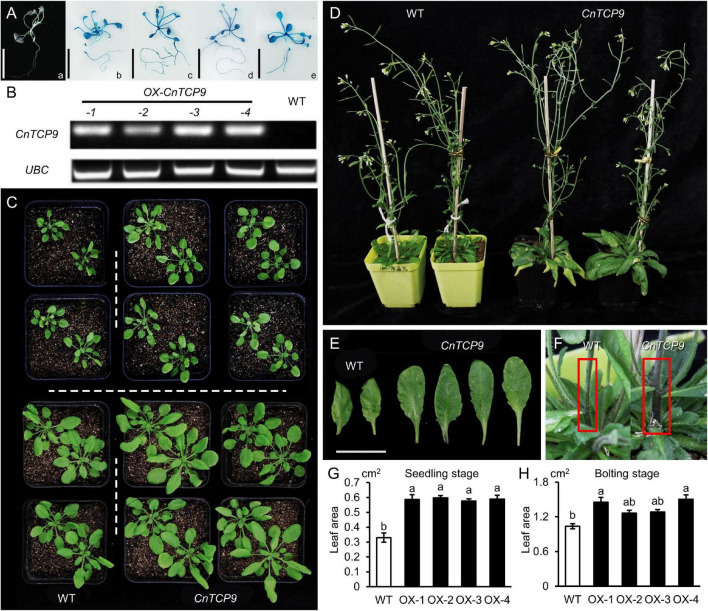
Phenotype of overexpressing *CnTCP9* in Arabidopsis. **(A)** GUS staining of T_3_ generation. (a) Non-transgenic Arabidopsis was used as the negative control. (b–e) Transgenic *CnTCP9* positive seedlings. **(B)** RT-PCR of *CnTCP9* in WT and *OE* (Overexpressing *CnTCP9* Arabidopsis). **(C)** Germination period and seedling period of WT and OE. **(D)** Mature period of WT and OE. **(E)** Leaves of WT and OE; bar = 2 cm. **(F)** The main stem of WT and OE. **(G,H)** Statistics of mature leaf area of WT and *OE*. Data are means ± *SD*. The different letters represent the values significantly different at *p* < 0.01 according to the one-way ANOVA test.

A total of 14 resistant T_1_ Arabidopsis seedlings *OE* (Overexpressing *CnTCP9* Arabidopsis) were screened by hygromycin screening. The seeds of the T_3_ generation of positive transgenic seedlings were collected and screened in hygromycin resistance medium, and further identified using GUS staining. At present, four homozygous Arabidopsis lines with a single copy of *CnTCP9* which show different phenotypes from the wild type have been identified (Figure 5A). The expression levels of *CnTCP9* in T_3_ transgenic plants and WT were shown in Figure 5B. No expression was observed in the WT Arabidopsis plants. At the early seedling stage, the transgenic lines showed an enlarged phenotype compared to that of the wild type. The enlarged plant was characterized by a greater plant height and longer leaf length. All four transgenic plants exhibited similar phenotypes (Figure 5C). In the mature period, the advantage of plant height was no longer obvious in transgenic lines (Figure 5D), but the difference in leaf length and leaf area was far more significant (Figures 5E,G,H). Moreover, stem thickness showed significant difference in the transgenic lines (Figure 5F). Significant phenotypic differences were found between overexpressed transgenic lines and wild-type *A. thaliana* that were mainly reflected in the rapid growth and leaf enlargement, indicating that overexpression of *CnTCP9* can indeed induce plant phenotypic variation.

**FIGURE 6:**
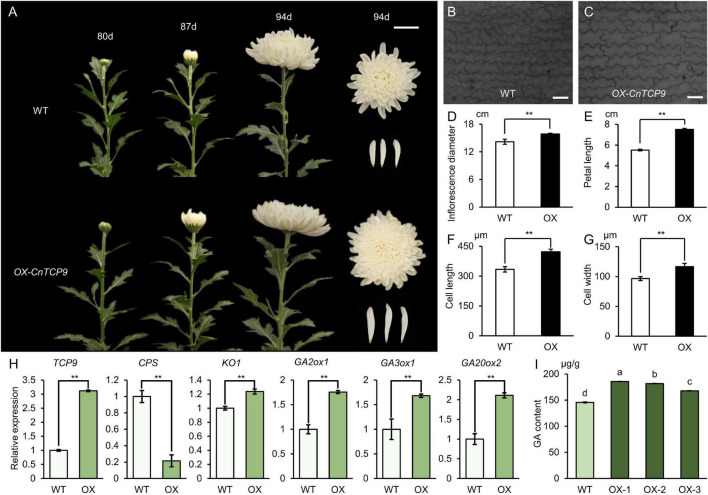
Phenotype of overexpressing *CnTCP9* in chrysanthemum. **(A)** Phenotypes of WT and *OX-CnTCP9* transgenic plants at 80 d, 87 d, and 94 d after planting in the field; bar = 5 cm. **(B,C)** Cells of the middle regions of petals at blooming stage in WT and *OX-CnTCP9* transgenic *C. morifolium* plants; bar = 170.0 μm. **(D)** Inflorescence diameter of WT and *OX-CnTCP9* transgenic plants. **(E)** Petal length of WT and *OX-CnTCP9* transgenic plants. **(F,G)** Statistics of petal cell length and width of WT and *OX* (Overexpressing *CnTCP9 C. morifolium*). Data are means ± *SD*. Two asterisks represent the values significantly different at *p* < 0.01 according to the *t*-test. **(H)** qRT-PCR analysis of GA pathway genes in WT and *OX*. Data are means ± *SD*. Two asterisks represent the values significantly different at *p* < 0.01 according to the *t*-test. **(I)** Statistics of GA3 content of WT and *OX*. Data are means ± *SD*. The different letters represent the values significantly different at *p* < 0.01 according to the one-way ANOVA test.

Due to the lack of a *C. nankingense* transgenic system, we transformed *CnTCP9* into ornamental varieties *C. morifolium* through *Agrobacterium*-mediated leaf disk transformation. Both the transgenic chrysanthemum and Arabidopsis showed similar phenotypes. The phenotypes of *OX-CnTCP9* and WT plants were observed at 80, 87, and 94 days after growth in the greenhouse under the same conditions. The *OX-CnTCP9* plants bloomed earlier than the WT plants (Figure 6A). After blooming, both the inflorescence diameter and petal length of *OX-CnTCP9* plants were distinctly larger than those of the WT plants (Figures 6D,E). By observing the length of petal cells under the optical microscope, we found that the increase of petals length was brought by the growth of cells length (Figures 6B,C,F). We also found the increased cells width in *OX-CnTCP9* plants petals (Figure 6G). These results showed that *CnTCP9* promoted petal cell development and increased flower size in chrysanthemum. It is widely believed that TCP transcription factors regulate plant development by interacting with other proteins or by binding to other gene promoters. To further explore the biological function of CnTCP9, we performed *Y2H* screening for searching interaction partner. The results showed that CnTCP9 could interacted with multiple hormone pathway proteins (Supplementary Table 5). Interestingly, we detected Gibberellin 3-beta-dioxygenase 4 (GA3ox4) as one of the interacting partners (Supplementary Table 5). GA3ox4 is a key enzyme in gibberellin synthesis, directly acting on the formation of active GAs, and plays a vital role in the growth and development of plant. Moreover, the GA-stimulated protein ubiquitin conjugating enzyme also have detected (Supplementary Table 5). This indicated more frequent connections between CnTCP9 and GA-related. The expression of GA pathway-related genes was also verified in the overexpressed transgenic lines, and the genes downstream of the GA pathway were all upregulated (Figure 6H). We further verified the content of GA (GA3) in transgenic chrysanthemum by HPLC experiments and found that the level of GA was significantly higher in *OX-CnTCP9* chrysanthemums plants compared to WT (Figure 6I).

## Discussion

TCP family members have been described in various species, like Arabidopsis, rice, *Medicago truncatula*, and *Gossypium barbadense* (Li, 2015; Wang et al., 2018; Zheng et al., 2018; Zhang et al., 2019b). In our study, we cloned 23 *CnTCP* genes based on the transcriptome sequencing of *C. nankingense*. Furthermore, all *CnTCP* genes were named according to their similarity, i.e., *CnTCP1*-*CnTCP18* and clustered two groups: Class I and Class II (Figure 1A). TCPs with similar motif compositions were clustered together. Similar to Arabidopsis and rice, CnTCP proteins number occupies a higher proportion in the PCF subfamily and CIN subfamily, and a lower proportion in the CYC/TB1 subfamily (Figure 1A).

RNA-seq and qRT-PCR data showed a strong correlation between them (Supplementary Figure 1). The RPKM-based expression pattern of *CnTCP18*/*16*/*9* showed similar trend with qRT-PCR data, and *CnTCP1*/*11* were not detected in both data (Figure 2 and Supplementary Table 6). With an increase in ploidy, the expression of the TCP family genes showed significant changes, especially in the PCF subfamily (Figure 2 and Supplementary Table 6). The PCF subfamily had the largest number of genes, the highest gene expression, and the greatest relative expression differences. The PCF subfamily proteins have been reported to show a high degree of functional redundancy, and thus phenotypic changes are usually observed only in higher-order mutants (Uberti-Manassero et al., 2012; Aguilar-Martinez and Sinha, 2013). Class I TCP factors can adjust plant height through GA-mediated pathways, and these Class-I TCPs either positively or negatively modulate cell proliferation and expansion depending on the organ involved (Kieffer et al., 2011; Daviere et al., 2014). *AtTCP14* and *AtTCP15* also participate in GA-dependent germination, flowering, and inflorescence stem elongation (Resentini et al., 2015; Lucero et al., 2017; Zhang et al., 2019a; Xu et al., 2020; Ferrero et al., 2021). *AtTCP20* and *AtTCP9*, more distantly related Class-I TCPs, inhibit JA biosynthesis by repressing the JA biosynthesis gene *LIPOXYGENASE2* (Danisman et al., 2012). Meanwhile, the *CYC*/*TB1* family had the lowest number of genes and the lowest expression, and two genes were not expressed at the seedling stage (Figure 2 and Supplementary Table 6). Class II CYC/TB1 proteins often play a key role in the floret and inflorescence development in plants. For example, *TaTB1* (the wheat *TB1* ortholog) regulates inflorescence architecture in bread wheat, and increased dosage of *TaTB1* promotes inflorescence branching (Yuan et al., 2009; Dixon et al., 2018; Zhao Y. et al., 2020). In this study, although the expression level of the CYC/TB1 subfamily was not high, the increase in ploidy increased the expression levels of *CnTCP3* and *CnTCP15* (Figure 2 and Supplementary Table 6), suggesting that the CYC/TB1 subfamily may also be sensitive to polyploidization. The CIN subfamily has also been widely reported to be related to leaf morphogenesis (Nath et al., 2003; Crawford et al., 2004; Lan and Qin, 2020). However, in this study, two high expressed CIN subfamily genes (*CnTCP2*, *CnTCP4-1*) did not differ significantly between diploid and tetraploid, while the expression of other significantly differentially expressed genes were low (RPKM < 10). We believed that it is implausible to consider differential expression of this subfamily as a key factor in phenotypic variation. The antagonistic effects of Class I and Class II TCPs on leaf senescence and cell production indicate that Class I and Class II TCP transcription factors can regulate the same genes and biological processes, act as either activator or suppressor (Danisman et al., 2012). After WGD, the opposite expression pattern of the PCF subfamily (Class I) and CYC/TB1 subfamily (Class II) could also imply an antagonistic relationship between Class I and Class II (Figure 2).

Heterologous expression of the *C. nankingense TCP* gene (*CnTCP4*) suppresses cell proliferation and reduces leaf size in *A. thaliana* (Qi et al., 2019). The results of this study showed that many *TCP* genes were differentially expressed between the diploid and tetraploid plants, while the expression level of *CnTCP4* did not differ after WGD. According to the transcriptome data, there was no increase in the number of *TCP* genes after the autopolyploidization of *C. nankingense*. The enlargement of tetraploid plant organs was caused by some specific *TCP* genes expression changes, such as *CnTCP9*. We found that the expression of *CnTCP9* changed dramatically after polyploidization. In both *A. thaliana* and *C. morifolium*, the *CnTCP9* transgenics produced larger leaves and stems, thicker inflorescence diameter, and larger petal lengths compared with those of the wild type (Figure 5). Larger and longer leaves facilitate light interception, which is advantageous for plant growth. Since the *attcp20* mutant did not show any obvious phenotypic differences, ectopic expression of the *AtTCP20* plant was generated, which was tagged with an EAR transcriptional repressor domain (Herve et al., 2009). Meanwhile, overexpression of *AtTCP20* showed pleiotropy, including small, wrinkled, or curled leaves, delayed inflorescence, aborted siliques, and few seeds (Herve et al., 2009). The different functions of *CnTCP9* and *AtTCP20* could due to differences in protein sequences (51.57% identity); the TCP family differentiated into different regulation modes among different species. In addition, if the role of the TCP family is over-enhanced or inhibited, plant growth will be adversely affected. Although AtTCP20 and CnTCP9 were adjacent in the evolutionary tree, their roles in leaf development were not consistent. The results of transgenic *A. thaliana* and *C. morifolium* with *CnTCP9* showed that the phenotype of the overexpressed lines was different from that of the wild type at the seedling stage (Figures 5, 6), indicating that the overexpression of *CnTCP9* could cause phenotypic variation in plants after autopolyploidization. The increase in plant height was not observed in the mature plants, which may be influenced by gravity. Overexpression of *OX-CnTCP9* in chrysanthemums provided earlier flowering and increased flower size phenotypes than WT plants (Figures 6A–G). This also provides a new idea for improving the economic output value of chrysanthemum.

According to the findings in Arabidopsis, Class I TCP transcription factors regulate leaf development through GA metabolism (Yamaguchi, 2008; Danisman et al., 2012; Aguilar-Martinez and Sinha, 2013). Furthermore, GA signaling is crucial to modulate organ size, for example, the GA treatment produces a significant increase in petal length (Li et al., 2015). Transcriptions in petals were observed in response to GA, and found a regulated homolog of *CYCLOIDEA-like 5* (*CYC5*), which encode a TCP-domain containing protein (Li et al., 2015). Functional analysis indicates that *GhCYC2*, *GhCYC3* and *GhCYC*4 regulate ray flower petal growth by affecting cell proliferation until the final size and shape of the petals is reached (Juntheikki-Palovaara et al., 2014). In our study, we found a significant increase in petal length of *OX-CnTCP9* (Figure 6E), which also led to an increase in inflorescence diameter (Figure 6D), and further observations revealed that this increase in growth was brought about by an increase in the length and width of petal cells (Figures 6F,G). The transcription changes were also observed in GA pathway genes after polyploidization, and most of the them were up-regulated (Figure 3 and Supplementary Table 7). This illustrates the tight connection between *CnTCP9* and the GA pathway in plant growth and development. The verification of the GA pathway refers to the reported synthetic process of the GA pathway, and key genes were selected to verify their expression (*CPSCPS, KO1, KAO1, GA2ox1, GA3ox1, GA20ox2*) (Yamaguchi, 2008). By verifying the expression of GA pathway genes in *CnTCP9* overexpression transgenic lines, it was found that the genes downstream of the pathway were upregulated, but the expression level of genes upstream of the GA pathway did not change significantly or was even downregulated (Figure 6H). The results were also consisted with transcriptome data analysis (Figure 3 and Supplementary Table 7). In *Y2H* screening, we found that CnTCP9 interact with GA pathways related proteins such as GA3ox4 (Supplementary Table 5). GA content even showed a significant increase in *OX-CnTCP9* compared with WT (Figure 6I). We speculated that CnTCP9 could activated the downstream genes of the GA pathway by interaction the GA synthesis pathway proteins to regulate the plant growth and development, resulting a larger phenotype. It is worth noting that in chrysanthemum commercial production, GA solution is often sprayed to induce homogenous flowering, and more compact stems. Here, we revealed and manipulated genetic factors that affect these aspects can reduce the use of chemicals in protected cultivation. This study presents a closer step toward that direction. We also screened interaction of CnTCP9 with other hormone pathway proteins (Supplementary Table 5), like JA, ethylene, and salicylic acid response proteins, WRKY transcription factor (WRKY1, 6, 7, 57) (Jiang et al., 2017). The interaction between CnTCP9 and JA signal response proteins may affect the changes in JA pathway-related gene expression after polyploidization (Figure 3 and Supplementary Table 7). Other interaction proteins, including ferritin, WD-40 repeat-containing protein, ubiquitin conjugating enzyme, and protein TIFY 10A were involved in leaf and flower development (Supplementary Table 5; Ingvardsen and Veierskov, 2001; Murgia et al., 2007; Pazhouhandeh et al., 2011; Hakata et al., 2012; Zhao L. et al., 2020). This led us to realize that GA may not be the only pathway by which *CnTCP9* affects plant development and further research was needed to understand the specific modulating mechanisms.

Altogether, these data suggest that the expression level of the TCP family changed dramatically after WGD, especially in the PCF subfamily, and played an important role in the regulation of the growth process. Moreover, the PCF subfamily member *CnTCP9* positively regulates the expression of downstream GA pathways and influences the development of plant phenotype in Arabidopsis and chrysanthemum. This provides evidence for the regulation of gene family and gene expression in plants after WGD, and this can be linked to phenotypic variation.

## Conclusion

In this study, transcriptome analyses were employed to obtain *TCP* genes and identify differentially expressed genes after polyploidization between diploid and tetraploid *C. nankingense*. Eighteen *CnTCP* genes were identified, and many of them showed different expression after WGD. The change of *CnTCP9* expression was the largest, being 3.66 folds higher. Although expressed in the nucleus, *CnTCP9* did not have transcriptional activation activity. The heterologous expression of *CnTCP9* in Arabidopsis results in an enlarged phenotype and better growth in both the seedling and mature stages. The overexpression of *CnTCP9* in chrysanthemum promotes flower development, increases flower size, and activates the expression of genes downstream of the GA pathway. These findings provide new insights into how autopolyploidization affects plant growth and development.

## Data Availability Statement

The datasets presented in this study can be found in online repositories. The name of the repository and accession number can be found below: NCBI; PRJNA810751.

## Author Contributions

HW and FC contributed to conception and design of the study. HW, FC, YG, and ZY organized the data. HW, ZY, CT, JH, ZW, and SL performed the statistical analysis. HW and ZY wrote the first draft of the manuscript. CT wrote sections of the manuscript. CT, HW, SC, FZ, JJ, ZG, LW, WF, and FC contributed to writing—review and editing. All authors contributed to manuscript revision, read, and approved the submitted version.

## Conflict of Interest

The authors declare that the research was conducted in the absence of any commercial or financial relationships that could be construed as a potential conflict of interest.

## Publisher’s Note

All claims expressed in this article are solely those of the authors and do not necessarily represent those of their affiliated organizations, or those of the publisher, the editors and the reviewers. Any product that may be evaluated in this article, or claim that may be made by its manufacturer, is not guaranteed or endorsed by the publisher.

## Footnotes

1
https://myhits.sib.swiss/cgi-bin/estscan

2
https://www.ncbi.nlm.nih.gov/orffinder/

3
https://www.megasoftware.net/

4
http://rsb.info.nih.gov/ij/
