# Supplementary material for: Expression Analysis of TCP Transcription Factor Family in Autopolyploids of Chrysanthemum nankingense
Source: Front Plant Sci. 2022 Jun 2;13:860956. doi: 10.3389/fpls.2022.860956 (PMC9201386; doi:10.3389/fpls.2022.860956)
Supplement: Supplementary file 1 [file Data_Sheet_1.docx]

Table S1. Summary of transcriptome data

| Samples | Total Raw Reads | Total Clean Reads | Total Clean Nucleotides (nt) | Q20 percentage | N percentage | GC percentage |
| --- | --- | --- | --- | --- | --- | --- |
| 2X | 53,720,166 | 51,622,828 | 4,646,054,520 | 98.37% | 0.00% | 45.05% |
| 4X | 53,514,300 | 51,462,400 | 4,631,616,000 | 98.45% | 0.00% | 44.38% |

Table S2. Summary of transcriptome assembly

|  | Sample | Total Number | Total Length(nt) | Mean Length(nt) | N50 | Total Consensus Sequences | Distinct Clusters | Distinct Singletons |
| --- | --- | --- | --- | --- | --- | --- | --- | --- |
| contig | 2X | 120,260 | 38,565,294 | 321 | 524 | - | - | - |
|  | 4X | 129,710 | 43,199,348 | 333 | 576 | - | - | - |
| unigene | 2X | 70,895 | 41,493,345 | 585 | 886 | 70,895 | 26,650 | 44,245 |
|  | 4X | 78,687 | 48,461,520 | 616 | 981 | 78,687 | 31,409 | 47,278 |

Table S3. Primers used in this study

| Note | Name | Sequence (5′-3′) |
| --- | --- | --- |
| Clone | CnTCP1-F | AATGGTGAACAACAAGAAAGATGG |
|  | CnTCP1-R | TGTGCCCCTTCAACTATGGACTC |
|  | CnTCP2-1/2/3-F | GCTTGGTATGGAGGTTGATGAA |
|  | CnTCP2-1/2/3-R | GCGATTTCCTTACTTTTTCTTCA |
|  | CnTCP3-F | GGCTAAATAAAAGATGTATCCCTC |
|  | CnTCP3-R | GGTAATAGTATGAACAAGTAGCAC |
|  | CnTCP4-1-R | TTCTCACTGTGTAGCAGCCATTG |
|  | CnTCP4-2/3-R | CTTTCCTGTTTTGTGAGAGG |
|  | CnTCP4-F | CACCAACACCAAACCCTCACCG |
|  | CnTCP5-F | GTTCAACAATAGTTCAGGAGGA |
|  | CnTCP5-R | TCTACCTCGTTGCCCTCTCAAT |
|  | CnTCP6-F | GCCTCAATCACCACCCAGTTTC |
|  | CnTCP6-R | CACCTAAACAGCCAAAAAACATCATAAG |
|  | CnTCP7-F | AACAACTGACGGCACAACTAACG |
|  | CnTCP7-R | TGCTGAGGCGGACCTTGAA |
|  | CnTCP8-F | GGAGCCAACAAACAACCACTAC |
|  | CnTCP8-R | AACCTACCACATTTCATTCACAACC |
|  | CnTCP9-F | CCAAAGTTTCAACCTTTTACACCTT |
|  | CnTCP9-R | CAGATGGAAAACCCGAAAAGC |
|  | CnTCP10-F | TTCACAAAATCGCAAAACCCTA |
|  | CnTCP10-R | GCCAGCGTGATGCGATTGAGAT |
|  | CnTCP11-F | TTTAGTTATTACACAATGTTTTCCTCA |
|  | CnTCP11-R | CTTACAACATCAGTCCAGGTCCAC |
|  | CnTCP12-F | AGTGTTTTTTTAGGTTTATGTGTGATTC |
|  | CnTCP12-R | TTAGTCGGCTTCTTACGAGTGG |
|  | CnTCP13-F | GCACAAGTAATGAGTCAAGTTAGATG |
|  | CnTCP13-R | AACATCAAGAAAAATAACACTCACTT |
|  | CnTCP14-F | ATGAAAGTAGGGTATGGAGGTAGATG |
|  | CnTCP14-R | TTTTTGTCGATCTGCCTTGA |
|  | CnTCP15-F | GTAGGTAGTAAAAGGAGTAAGG |
|  | CnTCP15-R | CCCCTTGACACTGACCTATTTGC |
|  | CnTCP16-F | GAAATCAAAAACCTAAAAATCATAAT |
|  | CnTCP16-R | ATACGACCCTTACTGCCTTAC |
|  | CnTCP17-F | TAGGGAATCAACTGGCAAATGG |
|  | CnTCP17-R | TTTTTTCATCCTCATACTTTCTCG |
|  | CnTCP18-1/2-F | CAACATTTTATCTCCACAGCCAT |
|  | CnTCP18-1/2-R | CACAAACACCTAACCGAAGTCTC |
| qRT-PCR | CnTCP1-QF | CAATCGAGCTTTTGGAGTGA |
|  | CnTCP1-QR | GCAGATCAAGACCAGCCAAT |
|  | CnTCP2-1/2/3-QF | GGACAAGGGGGTTACCATTT |
|  | CnTCP2-1/2/3-QR | CAATCTTTCGCGATTTCCTT |
|  | CnTCP3-QF | GATTGCAGCCCAACAAATTA |
|  | CnTCP3-QR | GAGAAAAGATCGAACCGGTAA |
|  | CnTCP4-1-QF | CAAGGTGATGATGAGGAGCA |
|  | CnTCP4-1-QR | ACTGTGTAGCAGCCATTGGA |
|  | CnTCP4-2/3-QF | AGGTGGGTTTTCAGGGTTTC |
|  | CnTCP4-2/3-QR | CCTGAATGATACAGCCATCG |
|  | CnTCP5-QF | CAACCACCAAATTCGCTCAT |
|  | CnTCP5-QR | TGAAGTGATCTCTACCTCGTTGC |
|  | CnTCP6-QF | GCAGGTGTGTTGAGGGATTT |
|  | CnTCP6-QR | TGCACCTAAACAGCCAAAAA |
|  | CnTCP7-QF | CGAGGAGTGATTTTGGACAGGT |
|  | CnTCP7-QR | TTGAAGCGAAGCAAGCAAGTT |
|  | CnTCP8-QF | GGATGTTGGCAGCTTTGAAT |
|  | CnTCP8-QR | TCATTTTGGTCTTCATCACCAC |
|  | CnTCP9-QF | GGGCCAGATGAGTTTTTCCT |
|  | CnTCP9-QR | TTTGAAGACTGCGGTTGTTG |
|  | CnTCP10-QF | GATTTACGGAAGGGCAATTT |
|  | CnTCP10-QR | AATCACGACTGATGGCTAGTTG |
|  | CnTCP11-QF | AGAGCGAGAGCACGAGAAAG |
|  | CnTCP11-QR | ACCATTAACTCCTGCCCAGA |
|  | CnTCP12-QF | TAAACCCATACCGGCCTATG |
|  | CnTCP12-QR | TCATCATATCAGTGCCGGATT |
|  | CnTCP13-QF | TCCAGTGAATTCGATCCAAAA |
|  | CnTCP13-QR | ATACGGGCAAAAACATCGAA |
|  | CnTCP14-QF | CCCTTCAGTCCAATTTATTGTC |
|  | CnTCP14-QR | TTTGGTTTTTCCTTGTGATGG |
|  | CnTCP15-QF | CCCTTTTGGAGAGCATCAAG |
|  | CnTCP15-QR | CATGCCTTCCCATGGTCTAC |
|  | CnTCP16-QF | AACAATAGTGGGTTGCATTTCA |
|  | CnTCP16-QR | CATGGTCTAAGAGTGGCTGCT |
|  | CnTCP17-QF | TCTCCACATGGACTCACGAA |
|  | CnTCP17-QR | TCACGTACAACCTCTTGTTTGG |
|  | CnTCP18-1/2-QF | TTAACTTCGGGGGATCAAAA |
|  | CnTCP18-1/2-QR | CCTTAATTCCGAGACGGTGT |
|  | CPS-F | ACAAAGGATGGGATTTGCTG |
|  | CPS-R | TTCTTGGCATCTTCCAGAATTT |
|  | KO1-F | GGTGGTGTTTCGCTTTTGTT |
|  | KO1-R | GCTTTCTGGTGGAGATGGAG |
|  | GA2ox1-F | CCGTTTGGCTATGGGAATAA |
|  | GA2ox1-R | TCCGGATACGGAGGATAGTG |
|  | GA3ox1-F | TTATCCAGCCTGTCCAGACC |
|  | GA3ox1-R | GAGACGGTGTTGGGTTCTGT |
|  | GA20ox2-F | TCATGGCACTTTCAAATGGA |
|  | GA20ox2-R | CATATCGGCTCGGTAATGCT |

Table S4. The accession no. or locus ID of all the TCP genes

| Number in Figure | Gene ID | Number in Figure | Gene ID |
| --- | --- | --- | --- |
| AtTCP1 | AT1G67260.1 | AtTCP7 | AT5G23280.1 |
| AtTCP10 | AT2G31070.1 | AtTCP8 | AT1G58100.1 |
| AtTCP11 | AT2G37000.1 | AtTCP9 | AT2G45680.1 |
| AtTCP12 | AT1G68800.1 | Os01g11550 | Os01g0213800 |
| AtTCP13-1 | AT3G02150.1 | Os01g55750.1 | Os01g0763200 |
| AtTCP13-2 | AT3G02150.2 | Os01g55750.2 | Os01g0763200 |
| AtTCP14 | AT3G47620.1 | Os01g55750.3 | Os01g0763200 |
| AtTCP15 | AT1G69690.1 | Os01g69980 | Os01g0924400 |
| AtTCP16 | AT3G45150.1 | Os02g42380 | Os02g0635800 |
| AtTCP17 | AT5G08070.1 | Os02g51280 | Os02g0747400 |
| AtTCP18 | AT3G18550.1 | Os03g49880 | Os03g0706500 |
| AtTCP19-1 | AT5G51910.1 | Os03g57190 | Os03g0785800 |
| AtTCP19-2 | AT5G51910.2 | Os04g11830 | Os04g0194600 |
| AtTCP2 | AT4G18390.1 | Os04g44440 | Os04g0526000 |
| AtTCP20 | AT3G27010.1 | Os05g43760 | Os05g0513100 |
| AtTCP21 | AT5G08330.1 | Os06g12230 | Os06g0226700 |
| AtTCP22 | AT1G72010.1 | Os07g05720 | Os07g0152000 |
| AtTCP23 | AT1G35560.1 | Os08g33530 | Os08g0432300 |
| AtTCP24-1 | AT1G30210.1 | Os08g43160 | Os08g0544800 |
| AtTCP24-2 | AT1G30210.2 | Os09g24480 | Os09g0410500 |
| AtTCP3 | AT1G53230.1 | Os09g34950 | Os09g0521300 |
| AtTCP4-1 | AT3G15030.1 | Os11g07460 | Os11g0175700 |
| AtTCP4-2 | AT3G15030.2 | Os12g02090 | Os12g0209000 |
| AtTCP5 | AT5G60970.1 | Os12g07480 | Os12g0173300 |
| AtTCP6 | AT5G41030.1 | Os12g42190 | Os12g0616400 |

Table S5. Summary of Y2H screening

| PROTEIN | FUNCTION | SEQUENCE | FREQUENCY |
| --- | --- | --- | --- |
| H2A superfamily | centrosome duplication | GGAGTACCATACGACGTACCAGATTACGCTCATATGACAAGTTTGTACAAAAAAGTTGGAAGGGGACAACTTTGTACAAAAAAGTTGGAATCTAAACACACACAAACACATCTTTTTAACTTTTAGCAATGGCACCAAAGGCAGCAGAGAAGAAACCTGCAGCGGAGAAGAAGCCAGCAGCAGAGAAAGCACCAAAGCCCAAAGCAGAGAAGAAGCTCCCAGCAAAGGACACGTCAGGAGACAAGAAGAAGAAGAGACACAAGAAGTCGGTTGAAACCTACAAGATCTACATCTTCAAGGTTTTGAAACAAGTGCATCCCGATATTGGGATCTCAAGCAAAGCAATGGGAATCATGAATTCCTTCATTAATGATATCTTTGAGAAATTGGCTGCTGAGAGTTCCAAGCTTGTTAGGTATAACAAGAAGAACACTCTTTCGAGCAGGGAGATTCAGACTGCTGTGAGATTGGTCTTGCCTGGTGAGTTGGCTAAGCATGCTGTTTCTGAAGGTACCAAGGCTGTCACCAAGTTTACCAGCGGTTAAGGAATTGTTTGGTTAGATCTGGAGTGTTTGGATGTTGGTTTAGGGTTAGGGTTTGATTTTATTATGTAGATCTGGAATATCGATGTCTTATCGATGTTTATGAATTGTATTTTGTTTTAGTTTATGTGAAATGAATATCTATCTTTGATTTCTGTCTAAAAAAAAAAAAAAAAAAAAAAAAAACCCCCCCTTTTTTTTAAAAAAGGGGGGCCCCCCCCCGGAAAAAAATTCAAAAATTGGAAAACCCCCCCAGTTTCAAAAAAA | 6 |
| TIM-phosphate-binding superfamily | Glycolytic pathway; Trichome morphogenesis | CCATACGACGTACCAGATTACGCTCATATGACAAGTTTGTACAAAAAAGTTGGCGATGAGCAGATGCTTAATGTTCAAAACAAGAACTCTTCTTACTTTGTTGAGTGGATCCCAAACAACGTGAAGTCCACTGTGTGTGATATCCCACCAACTGGTCTTAAGATGGCATCAACTTTCATTGGTAACTCGACTTCTATTCAAGAAATGTTCAGGCGTGTGAGTGAGCAGTTTACTGCTATGTTCAGGAGAAAGGCTTTCTTGCATTGGTACACTGGTGAAGGCATGGATGAGATGGAGTTTACTGAGGCCGAGAGCAACATGAACGATCTTGTGTCTGAGTATCAGCAGTACCAGGATGCGACTGCTGATGAGGAAGGAGAGTACGAGGAGGAAGAAGAGGAGTATGACGAGGCTTAAGCCATATTGATTACAATTAGTGGTTGTAATATCTACTATTTTTTCCAAACATTGTAGGAATAGGGAATTGATCAATGCATTTTGTTTAACTGGAAAAGGTTGAGCTGTAATTTGTCTATTTTGGATCATAAGTAATGTTTCTTATTTTGTCAAAAAAAAAAAAAAAAAAAAAAAACCCCCTTTTCTTGTAAAAAGGGGGGCCCCACGCGCAAAAAAACCCAAAATACAGAAAACCCCCGCAAGTTACACAA | 5 |
| ferredoxin | Photosynthesis, participate in redox reaction | CCATACGACGTACCAGATTACGCTCATATGACAAGTTTGTACAAAAAAGTTGGCTCTCATTCACTCACTCTCCAACCAACATCATTCACAGCAATGGCAGCAACCACAGCATCTCTCACAGGCACCATGGCCAGCACCACCTTCCTACGTCGCCAAAACACTACCACTACCTTCAAACCATCAACCCTTTTTGGACTTAAGTCCAACACACCCAACAAAGGTGGTGTCACCATGATGGCCATTCACAAAGTGACCCTTATCACCCCTGAAGGCAAAATTGAGTATGAGAACCCTCATGATGTGTACATTCTTGACCATTCTGAGGAAAATGGTGTTGAGTTACCATACTCATGCAGAGCTGGTTCTTGCTCTTCATGTGCTGGTAAAGTTATATCTGGCGAAGTTGACCAGTCTGATGGAAGCTTTCTTGATGATGATCAGATCAATTCTGGTTGGGTCTTGACTTGTGTTGCTTACCCTAAAGGTGATGTTGTTATTGAGACCCACAAGGAAGAAGAGCTTACTGCATAATTTTTTATACTTAATAGAACTTGAGAATAAGAAATTTGATGTGGTGATTGTTGGTCGTTTCATGTGTCTTTTATTATTAAAGATGTAATGTAATGGGTTATATTTAAGATTTGGTATTGTTGAAATTTTATCCTAGTCTCTAGACTTAATTGCTTATGTTTGTTTTACTAGTTTTTAAGTATTTGGTTATGTATGATTATAATCTGTTACGCTAAATATAAAAAATCCAAGTCAGTTACTAAGGATTTTTCGATGAAAAAAAAAAATAAAAAAAACCCCCATCTTCTCTGTACAAAGGGGTGCTCCAGCTGGCAAATAATCGAAAATACGAGAAACACCCCGCAGATTTCACAAA | 4 |
| WRKY transcription factor（WRKY1、6、7、57） | Defense mechanisms (bacteria and fungi); Jasmonic acid, ethylene, salicylic acid response | CATACGACGTACCAGATTACGCTCATATGACAAGTTTGTACAAAAAAGTTGGAGCAGCTTTTGAAAGTGCATTTATGGATAAACGTGCCTGCAACAATGGTGCTAACTACATGACAACAGCATCACCACCGCCTTCGCCTGAAAAACTTGTAATAAAGCCAACTAACCAGTGTAACCACTATCACCAGCCCCAGTTATCACCTCCAAATCCCCCTGAGATGATCCCATATCTCAATACCAACCTCAGTTTTAACAACTGGCACTTGGATGGTGCTACTATTCCACCTTCATTTTCCTACCCTTTAGCACAAGTTGGATTTATGGAGGATTGCCAACAACTTGAAATCCCAAATAACGTTGAAGATGAATTATTGCAAGTCTATTCACCACCTTTTATCTCCCCAGTCACCTCTGAATCAAACTACACCACAGATTGGGAAAGTTCACCATCATACGATTTCCCGGTTGACCCAGAAGATGTATTCCCTGATTTCATATTTAACAACTTGTTTCTTTTAGATGATTAGTCGTAGATAATGTTAGACGATTAGATTATTAGCCCAAACCCGATTGTGTGTTTGAGGTTCATTAGGTTTAGGGGTTATGGTAGCTTATTGTCAAAATTATGCTCTATTATAAATAAACATGCTACCCTATGTAATTGACAAGTCATTCAATCAATAAAACATTATTACGGAGTATTATCTTAAAAAAAAAAAAAAAAAAAACCCCATTTTTCTTGAAAAAAGGGTGGCCCCACCCGCAAAAAATCCAAAAAACGAAAAACCCCGCAAAGTACACAAATA | 4 |
| RING finger domain-containing protein | Ubiquitination modification of histone H2A; Cell mitosis; Negatively regulated transport RNA polymerase II promoter | CCATACGACGTACCAGATTACGCTCATATGACAAGTTTGTACAAAAAAGTTGGATCCAACTTTTTTGTACAAAGTTGTCCCCCGGTGTTGGAGTTGGATGAAGCCGAGAAGGAGTTACAAGAGGATATGGAATTGTTGGATGTTGATGATGATGATTTTGAACAAGTGGACAAGTTGTCGTTACCTTTAGTCTCCAAGTGGAACCTCCCTGATGGAACACCTGTAGATGGGTTAGAGGTACCCGTTTTGAAAGAGGATGATTTTATTACTGATTCAATGTATCGTGATGATATTGTTTTTGGGCCAAGAAAAGATCCCCCTCCTCACGTCTTTGATGATCCTCCTCCTCCTCCTCCTGCCCATGTTTGTTCCATCTGTTTCAAACATTGGACTCTTCTCCATCACTTTTGCTGTCTTCCTTGTGGGCATATATTTGGAGCTCAATGTATACAAAAATGGCTTCAAACTAACTTACGTGGGACGTGTCCTCAATGCAACGTCTCATGCTCATTGGAGGATCGTAGATCTCTTTATGCAACCACCCTTTGCATTCCCTATGCTAGTAAAGGTGTCGCAAATCCAGAGATATCCACTAGACGATTTTCCTTCACCCAATCAGGATTAATCGAATTCAGAAAGTTTGAAATTGGACGGCGGAATTTTGCTTTGAAAAAAGATCAGATGCGAAGAAACGGCTGGCTGGTGTTGCAGGAAGGAAAAATGATTTAATGAAACGTCGGACTGACTTATTGGACAGGAGGGCTAGTGTGCTAAATCGGGCTAAGGCATTGGAAACAGCGGGGTGATTCATTGTGTCGGGCAGATGCATTGCGATTACGGGCTGATGCATTGGGCCCGGTGGGCGGATGAATATCTACGCGAGCTAAAGCATGCGAAGAAGAGGGCTAAAGCCATTGAGTCAAAGGCTAGGCATATAATCAGCGGGTTAAGCATTGAAGTCAGATTGCACTATTTTTCTCGAAGCTGCGCTACCTTTAAAAGAAACACTATACATTGTGCCGCAGCAGCA | 4 |
| ferritin | Intracellular iron balance; Leaf development | CCATACGACGTACCAGATTACGCTCATATGACAAGTTTGTACAAAAAAGTTGGATTTAATCTCCTTTTGAACAAAACCGATGCGGATCTCGGTTACGTACCGAGGAAACCCATCGGTTTCGTTAAATTGAATGGAAGGTGTGGTGCGAGTGTGGGTGAAGAAAATGTGGTGGTGAGTAGCGGTGTTTTGTTTCAGCCTTTTGATGAGGTTAAGAAGGATGAGTTTATTGTTCCGATATCGCCTCAGATGTCGCTTGCTAGACAAAAGTATTTTGATGAGTGTGAAGCTGCTATTAATGAGCAGATTAATGTGGAATATAATGTCTCGTACGTGTACCATGCTCTGTATGCATATTTTGATAGGGACAACGTAGCTTTGAAGGGCTTTGCTAAATTCTTTAAGGAATCAAGTGATGAAGAAAGAGAACATGCTGAGAAGCTAATGAAATATCAGAATATGCGAGGTGGACGAGTGACATTGCACACTATTGTTACTCCCCCTTCTGAGTTTGAGCATGTAGAAAAGGGAGATGCTTTATATGCAATGGAGCTAGCATTGTCCTTGGAGAAACTAGTCAATGAAAAACTTCTTGCATTGCACGCGGTGGCTGATAGAAACAATGATCCCCAAATGGCAGACTTCATTGAGAGCGAATTTCTAGCTGAGCAGGTTGAAGCAATAAAGAAAATATCAGACTATGTCTCTCAACTGAGAAGAGTTGGGAAAGGCCATGGGGTCTGGCATTTTGATCAGATGCTTCTTGAAGCTGGCGTTGCTGCATAATCAAATATTGTTGCGTATTTTGCCTAGTTGTACGTTGTCTAGCATAATCATTTGTAATTGCTACGATTAGACTCTTGCTTTGTGTGTGGTCTTGCCTATAATATTAGACTTGTAGTACCTTCCTGGTTTCATATGATTAGGACCTTCATAAACAAATTAACACGCACTTTCTTGTACAAAGTGGGTGCTCCAGCTGCAATAATCGAAATACTGGAAATCCCGCAAGGTTCCAG | 4 |
| UBQ superfamily | Protein catabolism | CCATACGACGTACCAGATTACGCTCATATGACAGTTTGTACAAAAAAGTTGGACGGTGATATAAAAAAAAAATGTCCTGCAAATCTTCGTGAAAACATTGACCCCTAAGACAATAACCCTTGAAGTCAAATCCTCGGACACCATCGACAATGTTAAGGCCTTTATTCAAGACAAAGAAGGTATTCCACCGGATCAGCAGAGGTTGATATTTGCCGGAAAACCCCTTGAACATGGTCGTACTCTTGCTGACTACCACATCCTTAACCAGTCAACTCTTCATTTGGTCCTAAGGCTGAGGGGAGGAATGGGGAAAACTATACAGATTGAGCCTTATTTAAAGGGGTTGGCTAGGAAATCACACCTCTTATGGGTTGTATGCCTCCAATGTTGTGCCCATCTTCCCCCACTGGCTGGAAACTGATCGATAAACAATGGAGGGCCCATCAACCAACTTCCGCCCTTCAACATGATCGGGATGGATTGACTGTTTGGCGTGTTGGAAAGGAACTTGTCTCTTTATTTCTAGAACTTGTCTTGTTTTTAACTACATATTGAAAGTGAACTTGTTGAATTGTTTGTTTGAACTTGCTGAATTGTGTTGTTTATATTCACAGAAGCCTTCTTGATTTGATATCGTTAACGTCTAACATAGTTCATACCCTGTATTTCCGGCTAGATCAGCTACCCAGGGAAACTAAATGGATAAGTAATTCAAGACCCTCTTTTGTCTAAGTCTTCTTTAGGAAGAATATAAATAAGACCCGCACTTTTTTTGCAAAAGTGGCGCACTCAGTCGGCAAAATATCTTTAAAATGTGGAAAAACACCGAGATGATATGTTGGAATTCGGGAAGTTCCCTTTTCAAGAGGCCAACCCTTCATGACATACTACATACTCTCTTGTTGGACATAGTGT | 3 |
| WD-40 repeat-containing protein | Cell differentiation; Chromatin modification; DNA replication; Flower development; UV-B response | GGAGTACCATACGACGTACCAGATTACGCTCATATGACAAGTTTGTACAAAAAAGTTGGAGTGGTTGAAGATGTATCATGGCATTTGATGAACGATAGTTTGTTTGGATCTGTTGGGGATGACTGCAAGCTGATGATTTGGGACTTGCGCACAAATAAACATGAACAAGCTGTTGTAGTTCATGAGAAAGAGGTGAATTACTTATCTTTCAACCCATATAATGAGTGGGTTCTGGCTACAGCATCTTCAGATACCACTGTTGGTCTGTTTGACATGCGGAAGCTGACAGCACCTCTGCATGTTTTAAGTAGTCATACGGAGGAGGTGTTCCAAGTGGAGTGGGATCCAAACCACGAAACAGTGCTTGCATCCTCTGCTGATGACAGAAGGTTGATGGTTTGGGATCTAAACAGGGTCGGAGATGAACAGTTGGAAGGAGAAGCAGAGGATGGCCCTCCAGAGCTTCTCTTCTCTCATGGTGGTCACAAGGCCAAAATATCAGACTTCTCATGGAACAAGAACGAACCCTGGGTTATATCTAGTGTTGCTGAAGACAATGCTCTACAGGTCTGGCAAATGGCCGAAAGCATTTATCGCGAAGATGATGACATTTGAAGATCACCTTTGTACTCTTTGTTTATGAGTTAGAAAATTACAACTCTACTCTACTCAAAACTTGTATAGTGGTTGTTGTACCGAATATAACCTCTCTTCAGTCCCATATATGCTAGTAGGCCTGGGGCTTTTCAATTGGGTTTGTGCGTTTGTACAAGTAGTTTTACCTTGTTTACTTTATTAATGCATATTGTTAAGAAAAAAAAAAAAAAAAAAACCCCACTTTTCTTGTACAAGGGGGGCTCCGACCTGCAAAAGAATCTAAAAATCTGAAAACCCCCCAAATGTTACACAA | 3 |
| protein TIFY 10A | Defense mechanism; Flower development; Jasmonic acid signal response; Pollen development | CCATACGACGTACCAGATTACGCTCATATGACAAGTTTGTACAAAAAAGTTGGAGGCAAGGTTTATCTCCGGATCGGTCCAGGTCCGGTCAAGTCAACTTTTTCACCTACTTGTAATCTTTTTTCTCATTACTTGAAGGAGAATAATAATTTTCCAGATCTAAAACTTGGCAGAAGGGTACTGACACCATCATCAACAACTCCAACAATGAATCTATTTCCAATGGTTGAAACCCCACGCGCCACCATGCAGCAGCAACCAACAACAGCCAAGCAGCCAATGACTATATTTTATAATGGTCAAGTTGTTGTCTTTAATGACTTATCTCCTGAAAAAGTTGAAGAAATTATGAAAATTGCTGAAAGGGGAGCTGCTGCTCAAAAGAAGCCTGTTGTTAAAATACAAGAATGTTCAAAGAATGTTATTGTTGCTAATTCTGATCTTCCAATTGCTAAAAAAGCTTCCCTTGCTAGATTCTTGGAGAAGAGGAAAGACAGAATTACGGCTCTTTCGCCATACCGAGTTCCAGAATCTCCAGAGCAAGAGGATAGTAAGACATGGTTGGGACTAGGTGCACACGCTCATGTCCTATAATCTCCATTGATGGATCAAGCATATTGGTTACGATTAGGGTGGTCTTTCGTGTTACTTTCGTTTTTTTACGGATGGCCGCCGGTTTTATTTACTATATTATCTCTAACATATTTTTTGCATCTCTCTTGCCATGAAAGATGTATACTATATAATATGCACTTCGAATTACTTTTACCTTGTGATATGCAATA | 3 |
| chlorophyll a-b binding protein, chloroplastic-like | photosynthesis; Response to red and blue light; Fructose response | CCCATACGACGTACCAGATTACGCTCATATGACAAGTTTGTACAAAAAAGTTGGAAGTGACTACGGTTGGGACACTGCTGGGCTTTCTGCTGACCCAGAAACTTTTGCCAAGAACCGTGAGCTTGAGGTGATCCACTCAAGATGGGCCATGCTTGGAGCTCTTGGATGTGTTTTCCCCGAGCTTTTGGCCCGTAACGGGGTTAAGTTCGGTGAGGCTGTTTGGTTCAAGGCTGGAGCCCAAATCTTTAGCGAAGGTGGTCTTGACTACTTGGGTAACCCAAGCTTGGTCCATGCACAAAGCATTTTGGCCATATGGGCTACTCAAGTGGTCTTGATGGGCGCAGTTGAGGGTTACAGAATTGCTGGTGGCCCATTGGGTGAGGTTGTTGACCCACTTTACCCTGGTGGTAGCTTCGACCCATTGGGCCTTGCTGATGACCCTGAGGCATTTGCTGAGTTGAAGGTTAAGGAGCTCAAGAATGGAAGACTAGCTATGTTTTCAATGTTCGGATTCTTTGTTCAAGCCATTGTGACCGGAAAGGGACCTTTGGAGAACTTGGCTGATCACCTTGCTGACCCTGTTGCAAACAATGCTTGGTCATATGCCACCAACTTTGTTCCCGGAAAGTGAGATTAGAATGAGTGTGTGTGTGTGACATGAAGTTTATTGGTGTCATATTGTTTGTGAATTATATAATTAGTAATATATGGTTTAATTTTGGGCAAAAAAAAAAAAAAAAAAAAAAAAAAAAAAAAAAACCCCCTCTCTTTCTATAAAAAGGAGGGCGGCCACCCCCCAAAAAAAAACCCAAATATAAAAAACACCGCCCCGATTATAACAA | 3 |
| ubiquitin conjugating enzyme | Histone H2B ubiquitination; Leaf morphogenesis; Negative regulation of flower development; Protein catabolism; Vegetative reproductive meristem | CCATACGACGTACCAGATTACGCTCATATGACAAGTTTGTACAAAAAAGTTGGACAATTTAAAAATAATAATAACCAAATTTTTATAAAAATCTTTTTCATGACGCTTGGTTCAGGTGGATCTAGTGTTGTTGTACCTCGGAATTTCAGACTATTGGAGGAGCTTGAGCGTGGAGAGAAGGGTATTGGAGATGGTACTGTGAGCTATGGAATGGATGATGGTGATGACATTTATATGCGGTCTTGGACTGGTACCATCATTGGTCCTCACAATTCTGTACATGAAGGTCGGATCTATCAGTTGAAGCTGTTTTGTGACAAAGATTACCCTGAGAAGCCTCCTACTGTTCGTTTTCATTCTCGGGTTAACATGACTTGTGTCAACCACGAGACTGGAGTGGTGGACCCAAAGAAGTTTGGGATACTAGCGAATTGGCAAAGAGAGTACACGATGGAAGATATACTGACACAACTGAAGAAAGAAATGGCGGCCGCACACAACCGAAAACTTGTTCAGCCACCAGAAGGTACCTTTTTCTAGATGATGGATGAATGTTCTTAGATGATAGTTTGCATATTCTTTATGCAAAATTAATGGTTTGGATTTAGAGGATATGATACTTTGAAAACGCTTTTTAATTTGTATTTTGACTCTCATGGATGTTTCTGCATGCTGTTTGATTCATGTGATTCGTGTTTGTGTCAAATGTTTATTATGAATATATGAGACGTCATGGATGTTTATGCAAAAAAAAAAaAAAAAAAAAAAACCCCTTTTTTTCTTGTAAAAAGGGGGTCCCCACCTGAAAAAAAATCCAAAATACCTGAAACACCTCGCCAGTTCTACACAAA | 2 |
| prephenate dehydratase | Growth and development; Root hair elongation | GACGTACCAGATTACGCTCATATGACAAGTTTGTACAAAAAAGTTGGAGGGTTGAGAGATACCGCGGCGATTGCTTCTGCACGCGCCGCAGAGCTTTATAATTTGAACATCCTGGCTGATGGGATCCAGGATGATTCTAGTAATATCACGCGCTTTGTTATGTTAGCGCGTGAGCCTATTATTCCTCGGACGGACAGGCCGTTTAAGACTAGTATTGTTTTCGCGCACGATAAAGGGACTTCGGTTTTGTTTAAAGTATTGTCCGCGTTTGCTTTTCGGAATATTAGTTTGACTAAGATTGAAAGTAGGCCTCATCGAAACCGTCCCATAAGGCTTGTGGATGATGCAAATGTTGGTACAGCTAAGCATTTTGAGTATATGTTTTATGTCGATTTTGAGGCGTCTATGGCGGATGTTAGAGCCCAAAATGCATTGGCTGAGGTTCAGGAATTTACTTCTTTTTTGCGGGTTTTGGGAAGTTATCCTATGGATATGACTCCTTGGTCGCCTCCTAGAGACGATTAATCAGAATGTGACGTTATTTAATTTATGTATATTTGAAGGTTAAAAACCCGTTTTCATAAGTTTTAGGGTTTATTGTTACATAAAAAAAAAAAAAAAAAAAAACCCACCTTCTTTTGAAAAAGGGGGGCCCGACCCCCAAAAAAATCAAAAAAACCGAAAAACCCCCCATTTTACACAAA | 2 |
| MYB transcription factor（MYB9A） | Abscisic acid reaction | CCCATACGACGTACCAGATTACGCTCATATGACAAGTTTGTACAAAAAAGTTGGCTCTCTCTTTTATCTCTTTCCTCAAAACTCACACACTCAAAAGTGTGTGTTTGTGTGTGTACAAAAATACAAAACAAAAATGGGAAGAGCACCTTGTTGCTCAAAAGTTGGGTTACACCGAGGAGCATGGTCCAGTGAAGAAGATAAACTACTTACCGACTACATTCAAACTCATGGTGAAGGTCAATGGCGTAATTTGCCTACTAAAGCTGGGTTGCTAAGATGCGGGAAAAGCTGTAGACTAAGATGGATGAACTATCTTCGGCCCGGGATCAAGAGAGGAAGCTTCACCCAGGAAGAGGATGACCAAATTATCCAGCTTCATTCAGTCCATGGTAACAGGTGGTCATTCATAGCCACCGAACTCAAAGGCCGAACCGACAATGAGATTAAGAACCACTGGAATTCGCATCTTAAGCGTATAGCTGCGAATTCCAGTGGGGACCAAGAAGATGAACCCGAAACCAATAAAAGGCCTAAAAAGAAAAGAAGAAACACTAATAGCAAGTCTACTAAAGTCAAACCAGTTAACGTGCCCAAAAATATTGTACCTCAAGAAATTGTTCCTCAGCCTGCTTCATCGCATACTTCATTGTCTTCCTCGCAATCGGCCTCGTTTACTTTGCGTAAGACCGATAGTTTCGATAGCGTAATGATGAGCGGGTCATCGTCAAGTTCGAGTGTTGATCAAGGAGTAGAAGCTGATTTATTAGCCACGGATTTCTCGTGGCCTAGTTGGGCACCAATGTTCGAGATTGATCAAGGTATAGGAAGTACTATGAATGGTCTGGATGATCACGATTTACCATT | 2 |
| centrosome and spindle pole-associated protein 1-like | Positive regulation of cell division; Positive regulation of cytokinesis | CCATACGACGTACCAGATTACGCTCATATGACAAGTTTGTACAAAAAAGTTGGAAGGGGACAACTTTGTACAAAAAAGTTGGAAATTACGATTCTTAGAGGAAACCATGAAAGCCGGCAAATAACTCAAGTGTATGGCTTTTATGATGAGTGTTTGAGGAAGTATGGAAATGCAAATGTATGGAAACATTTTACCGACCTCTTTGATTATCTACCTCTGACTGCCCTAATTGAAAGTCAGGTTTTCTGTCTGCATGGTGGTCTGTCTCCATCTTTGGATACACTAGATAACATACGTGCTTTAGATCGCATACAAGAGGTTCCTCATGAGGGGCCAATGTGTGACCTCCTGTGGTCTGATCCTGATGACCGGTGTGGTTGGGGTATCTCTCCTCGTGGAGCTGGATACACCTTTGGACAAGATATCGCTGCACAATTCAACCATACAAATGGGCTCTCTCTTATTTCTAGGGCTCACCAGCTTGTTATGGAAGGTTACAATTGGTCTCAGGAGAACAATGTTGTAACTGTATTTAGTGCACCAAACTATTGCTATAGATGTGGAAATATGGCTGCAATACTAGAGATTGGAGAAAATATGGACAGAAATTTCTTACAATTTGATCCAGCGCCTCGTCAGGTTGAGCCCGAGGCAGCACGGAGAACTCCAGATTACTTCTTGTAAATCGGTGTTTGATAATATTACATTTCATCTTTTCATGTTGTTATAGTTCTTTCCCTTAGTCGAGAGAGAGTCCTCTATTGCGATTTCAAAGACAATGAAATTGATTGTTTCTTGTTCCAGTTAAAGATAGTTTTCTTGTTCCTCTGGC | 2 |
| eukaryotic translation initiation factor 5A-1 | Promote cell translation and elongation; Transcription termination; Translation code shift; Xylem development | CCATACGACGTACCAGATTACGCTCATATGACAAGTTTGTACAAAAAAGTTGGCACGCATTTTAATGACGACACCTCCTCAATCAAATATGTTAATTCTTCGGTAGTCGGTCTTCACCAATGTTTTGATACCGCCATACTCATTCACAAAGATAACATCCAAGGAAGTTTGTCTTTAATGGCACAAGAGTATAGCCAAAAAGCCTTAACAATAGCCAAAGGGAGTAGCATTCTTATCGATGACCACCCTTGTAAGGTTAGAAGTATTGAATACAGTGTAGCCAAAAGATACAAACCCAACGAATATACCTGTAAGTTTGATGCTTATGACATCTTTGATTTGACTACGTATACGGCACAAGCAAAAGCTACTGCATTGTGTATGTGTCCTTTTGTCCGCGACAATGTTTACCAGATTTGGCAGATCTCTAATGATGGACAAATGGTTGTGCAAAGTCTAGCAGATGGTACTTGGGGAAACATGACCCTGCCAGATCCAGGTTTGCTGCCTCATCCACATGTGCCTAATGCTGATGGAACTCCCGATCCCGATGAAGATGCTACTTTCATAACTCACTATCAGACGAAACTTCAAACGCGTAATCTGTTTCTGGAAGTAAAATATGTCATGGGAATGGAGCATATCATCGGTATCATGGAGCAATTGGAAGAACCGCCCCAGCCATCCGAGTCGTTGTACCATTAAGATTAGTTAGCATTTTACTTTCTGCACTACAATGATTAGTCCATTTGGATTTGGGTTTAGGAACAATTTAGTTTTGTGTACTTTAAAGATCTCTATCTTTGCTACAACTCAATGTAGTAACATCTCTCTTTTAAAATATTTCGTAATCAAAGTCATGTCTGTTTAAAAAAAAAAATAACACCACACTTTCTGTACAAGGGTGTCTCACGCTGCAGAATATACGATATTACTGAAACACCCCGCGAGTGATCACA | 2 |
| cyclin-dependent kinase F-1-like | Cell division; DNA intranuclear replication; Root meristem; mitosis; Negative regulation of cell cycle | GGAGTACCATACGACGTACCAGATTACGCTCATATGACAAGTTTGTACAAAAAAGTTGGCTTAAACGAGGAAGTTTGGCCCGGTTGTGCACAGCTTCCTGATTACCAAATAATTAACTTTGAAAAAGTGGAGAACCCAAGCGGTTTAGCTTCATGTCTTCCTAACCGATCCCAAGACGAGATTTCACTAGTAAAAAAACTGATTTGCTATGCTCCAGCGGCTCGGGCCACAGCAATGGAACTGCTCCATGATAAGTATCTAAATGAGGAACCGTTGCCTTTTCCTGTGTCCCAGCTGAGAGTTCCACTTTCGAACCATGGTACAGATGACTTCTCGTCTGGGTCTGAGGAATGGGGTGATCGCCGAGACTTGGGTTCAGACTCGGATTTTGATGAGTTTGATGTTACGACAGGAAGTGGTGGTGATTTTTCAATTCGTTTTTCTTAAGAAAGTGTGGCTATTGGTGATTCAGGTAAAATTTATTTACTATAAATTATTATTTTTATAATTCTTTGTAAATTTCAAGTGAAGATGTATGCGATGTGAAACAATTCAATTAAAGTACTTGGAAACCTTACAAATTTTGCGAGTTCTTGTATATGTATGTTTGGGAACTTCTTGTATATGTAAAATATGAAGGGAATTGAATGTAAGCTGGTTTCAGTAGAATCTGATATGGCTTCCCTTTTGGTTGCCGAAAAAAAAAAAAAAAAAAAAAAAAAAAACCCCCCCTTTTTTTTAAAAAAGGGGGGGCCCCCCCCCCAAAAAAACCAAAAAAACAAAAACCCCCCCCGATTACCCCCA | 1 |
| Gibberellin 3-beta-dioxygenase 4 | Gibberellin biosynthesis | CCATACGACGTACCAGATTACGCTCATATGACAAGTTTGTACAAAAAAGTTGGAAGGAATTTAGCTGAAGGTTTAGGCGTGAGAGGTGAAAATACTGGATTTGAGAATTGGCCATGTCAATTTCGGATCAATAAGTACAATTTTACCCCTGAAAGTGTTGGTTCCCCTGGTGTCCAATTACACACAGATTCCGGTTTTCTCACAATCCTTCAAGACGATGAAAGTGTTGGTGGTCTAGAAGTAATGGACAATTCTGGAAAGTTTTTAACCGTTGATCCTTGGCCAGATACCCTTCTAGTCAACCTTGGTGACATGGCAACGGTGTGGAGCAATGGGAGATTTTGTAATGTGAAGCATAGGGTACAATGCAAAGAAGCAAAGATACGTGTGTCGATAGCTTCGTTCCTTTTGGGGCCTAAAGGGATAGTGGAACCGCAATCAGAACTTGTGAACGATGACCATCCTTTTGTGTACATGCCTACCACCTACGAAGATTATAGGAAAATCAGGCTATCGACAAAGTTGCAGGCAGGGGAGGCTCTTGAGCATTTGTACACACCAAGTTTCAAGAAGTGATGAACCATGGTTGATCACATAGTGAATATTTTGAAGAATAAGTTTTATAGTTTGTTCCAAAATTATGCAAACTATATACATTTAATCCAGTGGAAATTCATGTCTCACATTGAGTTTGTATTTGATCAAAAACCAGTCTACTAATGGCATAGCACATGAGATCAATTTTAATAaAaaAAAAAAAAaAAAAACACCCACTTTCTCTTGTAACAAAGTGGTGTCCCACTGCAAATGATACTAAATACTGAAAAACCGCACAGTCTACACAA | 1 |
| basic leucine-zipper 44-like protein | Abscisic acid cells; Glucose cell corresponding | GGAGTACCATACGACGTACCAGATTACGCTCATATGACAAGTTTGTACAAAAAAGTTGGAACAAGGAAGAGGAAGCAACAACATCTAGATGATCTCACAGCTCAAGTGAGCCAACTCAGAAAGGAAAACAATCAAATCATATCAAGTGTGAGTATCACTACACAGCATTATGTCAGTGTAGAGGCTGAGAACTCGGTTCTAAGAGCCCAAGTGACTGAGCTCAGTAATCGCCTTCAGTCCCTTAACGAGATGATTGCTTTTATGTATCAACCTATGGACACTAATTGCGGCTTTGTGGACGAACAATATGGCAATGGGACTGAGTTTGTTGATGAGATGATGGTTAACTCACTGAGTTATCTTTGTGCTAACCAGCCCATTTTGGCTTCTGCTGATATGATTAAGTACTAAGTTAGACATGGGGTGGGGTGGGGTGGGGTGAGGAATAAAAGATTTGTGTAAAAAAGAAGATGAAGATTATGGCAAGGTCTTTGTGTACAAAGAGCTATATGATTGTAATGGGGTCTTTGTTAGATGGTAATATTGTCACTGATAATAATATTAATATTATGTTTGTTAAAAAAAAAAAAAAAAAAAAACCCCCCTTTTTTTTTAAAAAGGGGGGCCCCACCCGCAAAAAAATCCAAAAAACTGAAAACCCCCCCAAAGTTAACAAA | 1 |
| Cell elongation protein diminuto, putative | Brassinosteroid biosynthesis; Lignin Metabolism; Biological origin of secondary cell wall; Linear cell growth | CCATACGACGTACCAGATTACGCTCATATGACAAGTTTGTACAAAAAAGTTGGAGGGGACAACTTTGTACAAAAAAGTTGGAGGGGACAACTTTGTACAAAAAAGTTGGAGTACACTCTACCCTACGCATGGATAGGAAACTCTAGGAAACAATGTCCTGGAACATGCGCATATCCATTCGCAGTTCCGGACTACATTCCTGGATTAAAACCCGTAAAATCTCCCAACGGAGACGTGGCAATAGATGGGATGATAAGTGTAATAGCTCATGAACTAGCTGAAATAGCGTCTGACCCGTTTGCCAACGCTTGGTATGCTGGTCAAGACCCTGAGTTTCCAGTTGAGATAGCTGATTTATGTGAGGGTATTTATGGAACTGGTGGTGGTGGGTCCTACACAGGGCAAATGTTGAACGATGAAGATGGCGCCACGTATAATATGCATGGGATTAAACGTAGGTTTTTGGTTCAATGGGTTTGGAACCATGTGTTAAATTACTGTACTGGCCCTAATGCACTTGATTGAGAGGGTATAGTTTGTAATATTTTGTTTTGAGTTAACCATGGGCTGTTTAGTCTTTTGGTTGGTATTAAAAAAAAAAAAAAAAAAAAACCCACTCTCTTTGAACAAAGGGGGGCTCCACCCGCAAAAAAATCCAAAATACTGAAAAACCCCCCAAATTCACACAA | 1 |
| Dev-Cell-Death | Cell development and apoptosis | TACGACGTACCAGATTACGCTCATATGACAAGTTTGTACAAAAAAGTTGGCAAGAGGCAACTCTTTGGTTTACCACCCCGTTATCGCGACTCAGTGAGGCAAATCACCCCTGGTTTACCCCTTTTCCTGTACAATTATTCCACTCACCAACTTCATGGTGTCTTTGAGGCTGCAAGCTTTGGGGGTACAAACATTGATCCAACTGCTTGGGAGGACAAGAAAAATCAAGGCGAATCCCGCTTTCCTGCTCAGGTGCGAGTCATCACAAGGAATCTTTATGAGCCATTAGAGGAAGACTCATTCAGGCCCATTCTTCACCATTATGATGGACCTAAGTTCCGCCTTGAACTCAACATTCCAGAGGCTCTCTCACTTTTGGACATATTCGAAGAGAAGAAAAACTGAAGAGCCAATGAGTTAAGGGGTTTTCGGCACGCATGAAAGACATACACAAAATGATACTTGCCCAGGATTTTTTTTTCTTCTTTTGGTTTGCGAGGCGTAGGGTTATCTATTTATAATCGAGCTGAAAGGTGTTTTAGGTACGTTGTAAATATATATGGTACTCAAGGAATGTTGCTAGTGCTACTATCCAAATAAACAAACTTTTCGCTTGAATTCTTTGCAGTACTAATTAAGATCGAACATAAACAATTATCAAAAAAAAAAAAAAAAAAAAACCCACTCTCTCTGTACAAAGTGGGTGCCCCACCTCCAAAAGAATCCAAAATACTGAAAAACCCCGCGAGTTCAACAA | 1 |
| transcription factor E2FA-like | Cell cycle; Positive regulation of meiotic cell cycle; Positive regulation of DNA transcription | CCATACGACGTACCAGATTACGCTCATATGACAAGTTTGTACAAAAAAGTTGGGGCAGAAGTTCAAAAACTTTCTATGGAAGAAAATAGATTAGATGAACGCATCAGGGAAATGCAGGAAAAAATGAGGGACATGAGCGAAGATAACGCAAACCAAAAATGGCTTTTTGTGACCGAAGATGACATAAAAGGGTTACCGTGCTTTCAGAACGAAACCTTGATAGCAATTAAGGCTCCTCATGGGACCACTCTTGAAGTTCCGGACCCTGATGAGGCTGTTGATTATCCACAACGGAGGTACAGGATAATCCTTCGAAGCACAATGGGCCCTATTGATGTATATCTTGTCAGTCAATTTGAAGAAAAGTTTGAGGATGCTAATGGGGCTGACCAAACTATGGTGGCACCAGTTGCTTCTTGTTCTGGCTCAGATAATAATCCAGAGGTGACTATTGAACCACAGCCACAACATGCTCACAGTACAAACACTGATACTAATTTACCTGAAGACTCACCTAGTAGGATTATGAGGATTGTTCCTTCAGATTCTGATAATAACGAAGACTATTGGCTCCTATCAGATAGAGAAGTTCCTCTGACGGATATTTGGAACACAGAGTTACCTGAATGGGACGGAGTTGATTTGCTTAGTGAAGAATTTGGGTTGGCAGAAGTCGGGACACCAAGGGCTAACACTCTGCCATCTGGTGCTCCCGCTGCACCAGTAAATGGTGCACCCAGGTGAGCTAAGGTCAACCAGAATCGACAAATGGCTCAACACCATCGGCACTGAGAAACTGTTGTTTTTTCATACCGATTGCCATGAGGATACAATGAGACTGAAATTCAGATACAAAGCACAGCAGGTTTGTTGTACAGTAGAACAAGAAACATATTTCCATTCAAGCTGTAGGGATGAC | 1 |

Table S6. Fold change of *CnTCP* genes

| TCP-ID | UnigeneID | 2x_RPKM | 4x_RPKM | Fold Change |
| --- | --- | --- | --- | --- |
| TCP1 | Unigene21978_All | 0 | 0 |  |
| TCP2 | Unigene14138_All | 31.5357 | 36.1849 | 1.147421893 |
| TCP3 | Unigene2539_All | 1.7804 | 2.5721 | 1.444425733 |
| TCP4-1 | CL4533.Contig3_All | 9.1852 | 9.2955 | 1.012007141 |
| TCP4-2/3 | CL4533.Contig2_All | 5.975 | 5.1968 | 0.869757322 |
| TCP5 | Unigene19622_All | 5.0259 | 2.9106 | 0.579203883 |
| TCP6 | Unigene17038_All | 8.9494 | 6.5607 | 0.733118073 |
| TCP7 | Unigene6417_All | 37.2537 | 46.035 | 1.235716184 |
| TCP8 | CL15057.Contig1_All | 15.1924 | 18.2308 | 1.199981571 |
| TCP9 | Unigene25230_All | 20.9838 | 60.7537 | 2.895176509 |
| TCP10 | Unigene17131_All | 43.8351 | 30.8276 | 0.70326968 |
| TCP11 | Unigene31050_All | 0 | 0 |  |
| TCP12 | Unigene5654_All | 34.7114 | 14.5216 | 0.418369228 |
| TCP13 | Unigene14141_All | 1.948 | 3.1644 | 1.624114931 |
| TCP14 | CL9720.Contig2_All | 2.9369 | 4.7381 | 1.613090983 |
| TCP15 | Unigene17765_All | 0.3536 | 1.2309 | 3.474055274 |
| TCP16 | Unigene16806_All | 7.6196 | 23.9273 | 3.13994961 |
| TCP17 | Unigene10966_All | 1.8723 | 0.8501 | 0.454331928 |
| TCP18 | Unigene22759_All | 4.3901 | 21.7559 | 4.954772153 |

Table S7. Unigenes in hormone signals

| Hormone signaling pathway | gene | unigene | foldchange | pvalue | FDR | Function prediction |
| --- | --- | --- | --- | --- | --- | --- |
| *GA* | *CPS* | *cl16653.contig1_all* | 0.0718999 | 3.70E-123 | 9.40E-122 | (o04408\|ksa_pea : 491.0) Ent-kaurene synthase A, chloroplast precursor (EC 5.5.1.13) (Ent-copalyl diphosphate synthase) (KSA) - Pisum sativum (Garden pea) & (at4g02780 : 476.0) Catalyzes the conversion of geranylgeranyl pyrophosphate (GGPP) to copalyl pyrophosphate (CPP) of gibberellin biosynthesis; GA REQUIRING 1 (GA1) |
|  |  | *cl16653.contig2_all* | -0.140744 | 3.58E-12 | 1.36E-11 | (at4g02780 : 299.0) Catalyzes the conversion of geranylgeranyl pyrophosphate (GGPP) to copalyl pyrophosphate (CPP) of gibberellin biosynthesis; GA REQUIRING 1 (GA1) |
|  |  | *unigene35661_all* | 0.202261 | 2.96E-69 | 4.49E-68 | (o04408\|ksa_pea : 254.0) Ent-kaurene synthase A, chloroplast precursor (EC 5.5.1.13) (Ent-copalyl diphosphate synthase) (KSA) - Pisum sativum (Garden pea) & (at4g02780 : 228.0) Catalyzes the conversion of geranylgeranyl pyrophosphate (GGPP) to copalyl pyrophosphate (CPP) of gibberellin biosynthesis; GA REQUIRING 1 (GA1) |
|  | *KO1* | *cl15326.contig1_all* | 3.6439753 | 6.60E-214 | 2.90E-212 | (at5g25900 : 539.0) Encodes a member of the CYP701A cytochrome p450 family that is involved in later steps of the gibberellin biosynthetic pathway.; GA requiring 3 (GA3); FUNCTIONS IN: oxygen binding |
|  |  | *cl15326.contig2_all* | -0.334931 | 6.97E-34 | 5.79E-33 | (at5g25900 : 144.0) Encodes a member of the CYP701A cytochrome p450 family that is involved in later steps of the gibberellin biosynthetic pathway.; GA requiring 3 (GA3); FUNCTIONS IN: oxygen binding |
|  |  | *cl15326.contig3_all* | 0.3187196 | 2.78E-69 | 4.21E-68 | (at5g25900 : 575.0) Encodes a member of the CYP701A cytochrome p450 family that is involved in later steps of the gibberellin biosynthetic pathway.; GA requiring 3 (GA3); FUNCTIONS IN: oxygen binding; INVOLVED IN: gibberellin biosynthetic process, gibberellic acid mediated signaling pathway, ent-kaurene oxidation to kaurenoic acid |
|  | *KAO1* | *unigene5749_all* | 0.4074341 | 4.36E-08 | 1.26E-07 | (loc_os10g23180.1 : 462.0) no description available & (at2g32440 : 282.0) ent-kaurenoic acid hydroxylase (KAO2); ent-kaurenoic acid hydroxylase 2 (KAO2); CONTAINS InterPro DOMAIN/s: Cytochrome P450 |
|  |  | *unigene17101_all* | 0.1743684 | 6.68E-14 | 2.80E-13 | (at1g05160 : 280.0) Encodes an ent-kaurenoic acid hydroxylase, a member of the CYP88A cytochrome p450 family.; "cytochrome P450, family 88, subfamily A, polypeptide 3" (CYP88A3); CONTAINS InterPro DOMAIN/s: Cytochrome P450 |
|  |  | *unigene26922_all* | 0.5981735 | 0.000117 | 0.000242 | (at1g05160 : 149.0) Encodes an ent-kaurenoic acid hydroxylase, a member of the CYP88A cytochrome p450 family.; "cytochrome P450, family 88, subfamily A, polypeptide 3" (CYP88A3); CONTAINS InterPro DOMAIN/s: Cytochrome P450 |
|  |  | *cl7975.contig3_all* | 0.2556729 | 2.59E-09 | 8.21E-09 | (at2g32440 : 183.0) ent-kaurenoic acid hydroxylase (KAO2); ent-kaurenoic acid hydroxylase 2 (KAO2); CONTAINS InterPro DOMAIN/s: Cytochrome P450 |
|  | *GA20ox* | *cl15725.contig1_all* | 0.9381856 | 1.24E-68 | 1.86E-67 | (at5g51810 : 415.0) Encodes gibberellin 20-oxidase. Involved in gibberellin biosynthesis. Up-regulated by far red light in elongating petioles. Not regulated by a circadian clock.; gibberellin 20 oxidase 2 (GA20OX2) |
|  |  | *cl15725.contig2_all* | 0.5806903 | 6.54E-08 | 1.87E-07 | (at5g51810 : 123.0) Encodes gibberellin 20-oxidase. Involved in gibberellin biosynthesis. Up-regulated by far red light in elongating petioles. Not regulated by a circadian clock.; gibberellin 20 oxidase 2 (GA20OX2) |
|  |  | *unigene12251_all* | 1.272844 | 3.90E-111 | 9.00E-110 | (o04705\|gao1d_wheat : 97.8) Gibberellin 20 oxidase 1-D (EC 1.14.11.-) (Gibberellin C-20 oxidase 1-D) (GA 20-oxidase 1-D) (Ta20ox1D) (TaGA20ox1-D) (Protein Wga20) - Triticum aestivum (Wheat) & (at1g60980 : 95.1) gibberellin 20-oxidase 4 (GA20OX4) |
|  |  | *unigene23816_all* | -0.148751 | 9.13E-09 | 3.12E-08 | (at4g23340 : 237.0) 2-oxoglutarate (2OG) and Fe(II)-dependent oxygenase superfamily protein; FUNCTIONS IN: oxidoreductase activity |
|  |  | *unigene28242_all* | -0.040127 | 2.63E-07 | 7.13E-07 | (o04705\|gao1d_wheat : 147.0) Gibberellin 20 oxidase 1-D (EC 1.14.11.-) (Gibberellin C-20 oxidase 1-D) (GA 20-oxidase 1-D) (Ta20ox1D) (TaGA20ox1-D) (Protein Wga20) - Triticum aestivum (Wheat) & (at5g51810 : 137.0) Encodes gibberellin 20-oxidase. Involved in gibberellin biosynthesis. Up-regulated by far red light in elongating petioles. Not regulated by a circadian clock.; gibberellin 20 oxidase 2 (GA20OX2) |
|  |  | *unigene39677_all* | 0.1778567 | 2.48E-06 | 6.16E-06 | (at4g25420 : 117.0) Encodes gibberellin 20-oxidase that is involved in the later steps of the gibberellin biosynthetic pathway. Regulated by a circadian clock. Weak expression response to far red light.; GA20OX1 |
|  | *GA3ox* | *unigene18112_all* | -0.181025 | 9.47E-06 | 2.21E-05 | (at1g80340 : 359.0) Encodes a protein with gibberellin 3 &#946;-hydroxylase activity. The protein was heterologously expressed in E. coli and shown to catalyze the hydroxylation of both GA9 and GA20.; gibberellin 3-oxidase 2 (GA3OX2) |
|  | *GA2ox* | *cl16104.contig1_all* | -0.026035 | 1.27E-08 | 4.31E-08 | (at1g30040 : 155.0) Encodes a gibberellin 2-oxidase that acts on C-19 gibberellins. AtGA2OX2 expression is responsive to cytokinin and KNOX activities.; gibberellin 2-oxidase (GA2OX2) |
|  |  | *cl16104.contig2_all* | -0.365496 | 2.36E-27 | 1.64E-26 | (q9sq80\|g2ox1_pea : 151.0) Gibberellin 2-beta-dioxygenase 1 (EC 1.14.11.13) (Gibberellin 2-beta-hydroxylase 1) (Gibberellin 2-oxidase 1) (GA 2-oxidase 1) (Protein SLENDER) - Pisum sativum (Garden pea) & (at1g30040 : 144.0) Encodes a gibberellin 2-oxidase that acts on C-19 gibberellins. AtGA2OX2 expression is responsive to cytokinin and KNOX activities.; gibberellin 2-oxidase (GA2OX2) |
|  |  | *unigene9807_all* | 0.2565967 | 7.50E-272 | 5.40E-270 | (at1g02400 : 150.0) Encodes a gibberellin 2-oxidase that acts on C19 gibberellins but not C20 gibberellins.; gibberellin 2-oxidase 6 (GA2OX6) |
|  |  | *unigene13271_all* | 1.0278618 | 1.04E-38 | 1.09E-37 | (q9sq80\|g2ox1_pea : 92.8) Gibberellin 2-beta-dioxygenase 1 (EC 1.14.11.13) (Gibberellin 2-beta-hydroxylase 1) (Gibberellin 2-oxidase 1) (GA 2-oxidase 1) (Protein SLENDER) - Pisum sativum (Garden pea) & (at2g34555 : 83.6) Encodes a gibberellin 2-oxidase that acts on C19 gibberellins to deactivate them.; gibberellin 2-oxidase 3 (ATGA2OX3) |
|  |  | *unigene25509_all* | 0.0934237 | 1.00E-105 | 2.20E-104 | (q9sq80\|g2ox1_pea : 208.0) Gibberellin 2-beta-dioxygenase 1 (EC 1.14.11.13) (Gibberellin 2-beta-hydroxylase 1) (Gibberellin 2-oxidase 1) (GA 2-oxidase 1) (Protein SLENDER) - Pisum sativum (Garden pea) & (at1g78440 : 183.0) Encodes a gibberellin 2-oxidase that acts on C19 gibberellins.; Arabidopsis thaliana gibberellin 2-oxidase 1 (ATGA2OX1) |
|  |  | *unigene46936_all* | 0.4835708 | 4.23E-12 | 1.80E-11 | (q9sq80\|g2ox1_pea : 94.7) Gibberellin 2-beta-dioxygenase 1 (EC 1.14.11.13) (Gibberellin 2-beta-hydroxylase 1) (Gibberellin 2-oxidase 1) (GA 2-oxidase 1) (Protein SLENDER) - Pisum sativum (Garden pea) & (at1g78440 : 80.5) Encodes a gibberellin 2-oxidase that acts on C19 gibberellins.; Arabidopsis thaliana gibberellin 2-oxidase 1 (ATGA2OX1) |
| *JA* | *LOX1-6* | *unigene5327_all* | 3.6510668 | 2.52E-21 | 1.44E-20 | (at1g17420 : 81.3) Lipoxygenase; lipoxygenase 3 (LOX3); FUNCTIONS IN: oxidoreductase activity, acting on single donors with incorporation of molecular oxygen, incorporation of two atoms of oxygen, lipoxygenase activity, iron ion binding, metal ion binding; INVOLVED IN: growth, response to fungus, jasmonic acid biosynthetic process, response to wounding, defense response |
|  |  | *unigene6030_all* | -0.564891 | 1.34E-64 | 2.26E-63 | (at1g55020 : 376.0) lipoxygenase, a defense gene conferring resistance Xanthomonas campestris; lipoxygenase 1 (LOX1) |
|  |  | *unigene11108_all* | 0.0624327 | 4.56E-11 | 1.83E-10 | (at3g22400 : 847.0) LOX5; FUNCTIONS IN: oxidoreductase activity, acting on single donors with incorporation of molecular oxygen, incorporation of two atoms of oxygen, lipoxygenase activity, iron ion binding, metal ion binding; INVOLVED IN: root development |
|  |  | *unigene22590_all* | -1.113379 | 1.49E-06 | 4.22E-06 | (at1g55020 : 305.0) lipoxygenase, a defense gene conferring resistance Xanthomonas campestris; lipoxygenase 1 (LOX1) |
|  |  | *unigene23097_all* | 0.0586797 | 1.78E-05 | 4.50E-05 | (at3g22400 : 298.0) LOX5; FUNCTIONS IN: oxidoreductase activity, acting on single donors with incorporation of molecular oxygen, incorporation of two atoms of oxygen, lipoxygenase activity, iron ion binding, metal ion binding; INVOLVED IN: root development |
|  |  | *unigene42943_all* | -2.889396 | 3.90E-228 | 2.30E-226 | (at1g17420 : 520.0) Lipoxygenase; lipoxygenase 3 (LOX3); FUNCTIONS IN: oxidoreductase activity, acting on single donors with incorporation of molecular oxygen, incorporation of two atoms of oxygen, lipoxygenase activity, iron ion binding, metal ion binding; INVOLVED IN: growth, response to fungus, jasmonic acid biosynthetic process, response to wounding, defense response |
|  |  | *unigene43098_all* | -2.015212 | 2.86E-29 | 2.39E-28 | (q7xv13\|lox5_orysa : 142.0) Putative lipoxygenase 5 (EC 1.13.11.12) - Oryza sativa (Rice) & (loc_os04g37430.1 : 142.0) no description available & (at1g17420 : 137.0) Lipoxygenase; lipoxygenase 3 (LOX3); FUNCTIONS IN: oxidoreductase activity, acting on single donors with incorporation of molecular oxygen, incorporation of two atoms of oxygen, lipoxygenase activity, iron ion binding, metal ion binding; INVOLVED IN: growth, response to fungus, jasmonic acid biosynthetic process, response to wounding, defense response |
|  |  | *unigene43315_all* | -1.709688 | 6.92E-29 | 5.72E-28 | (q7xv13\|lox5_orysa : 104.0) Putative lipoxygenase 5 (EC 1.13.11.12) - Oryza sativa (Rice) & (loc_os04g37430.1 : 104.0) no description available & (at1g17420 : 100.0) Lipoxygenase; lipoxygenase 3 (LOX3); FUNCTIONS IN: oxidoreductase activity, acting on single donors with incorporation of molecular oxygen, incorporation of two atoms of oxygen, lipoxygenase activity, iron ion binding, metal ion binding; INVOLVED IN: growth, response to fungus, jasmonic acid biosynthetic process, response to wounding, defense response |
|  |  | *unigene49226_all* | 0.9997115 | 3.64E-15 | 1.82E-14 | (at3g45140 : 108.0) Chloroplast lipoxygenase required for wound-induced jasmonic acid accumulation in Arabidopsis.Mutants are resistant to Staphylococcus aureus and accumulate salicylic acid upon infection.; lipoxygenase 2 (LOX2); FUNCTIONS IN: lipoxygenase activity; INVOLVED IN: in 7 processes |
|  |  | *unigene49641_all* | 1.2720232 | 9.33E-06 | 2.44E-05 | (q8gsm2\|lox23_horvu : 160.0) Lipoxygenase 2.3, chloroplast precursor (EC 1.13.11.12) (LOX2:Hv:3) - Hordeum vulgare (Barley) & (loc_os02g10120.1 : 155.0) no description available & (at3g45140 : 144.0) Chloroplast lipoxygenase required for wound-induced jasmonic acid accumulation in Arabidopsis.Mutants are resistant to Staphylococcus aureus and accumulate salicylic acid upon infection.; lipoxygenase 2 (LOX2); FUNCTIONS IN: lipoxygenase activity; INVOLVED IN: in 7 processes |
|  |  | *cl507.contig1_all* | -0.210428 | 0.000885 | 0.001653 | (at1g72520 : 268.0) PLAT/LH2 domain-containing lipoxygenase family protein; FUNCTIONS IN: oxidoreductase activity, acting on single donors with incorporation of molecular oxygen, incorporation of two atoms of oxygen, lipoxygenase activity, iron ion binding, metal ion binding; INVOLVED IN: growth, jasmonic acid biosynthetic process, response to wounding, defense response |
|  |  | *cl507.contig2_all* | -0.08025 | 3.15E-14 | 1.34E-13 | (at1g72520 : 954.0) PLAT/LH2 domain-containing lipoxygenase family protein; FUNCTIONS IN: oxidoreductase activity, acting on single donors with incorporation of molecular oxygen, incorporation of two atoms of oxygen, lipoxygenase activity, iron ion binding, metal ion binding; INVOLVED IN: growth, jasmonic acid biosynthetic process, response to wounding, defense response |
|  |  | *cl507.contig6_all* | 0.7244369 | 1.64E-10 | 5.64E-10 | (at1g17420 : 1261.0) Lipoxygenase; lipoxygenase 3 (LOX3); FUNCTIONS IN: oxidoreductase activity, acting on single donors with incorporation of molecular oxygen, incorporation of two atoms of oxygen, lipoxygenase activity, iron ion binding, metal ion binding; INVOLVED IN: growth, response to fungus, jasmonic acid biosynthetic process, response to wounding, defense response |
|  |  | *cl507.contig7_all* | -0.745037 | 9.96E-06 | 2.32E-05 | (at1g72520 : 589.0) PLAT/LH2 domain-containing lipoxygenase family protein; FUNCTIONS IN: oxidoreductase activity, acting on single donors with incorporation of molecular oxygen, incorporation of two atoms of oxygen, lipoxygenase activity, iron ion binding, metal ion binding; INVOLVED IN: growth, jasmonic acid biosynthetic process, response to wounding, defense response |
|  |  | *cl847.contig1_all* | -1.894279 | 0.001892 | 0.003349 | (at3g45140 : 385.0) Chloroplast lipoxygenase required for wound-induced jasmonic acid accumulation in Arabidopsis.Mutants are resistant to Staphylococcus aureus and accumulate salicylic acid upon infection.; lipoxygenase 2 (LOX2); FUNCTIONS IN: lipoxygenase activity; INVOLVED IN: in 7 processes |
|  |  | *cl847.contig2_all* | -0.045723 | 3.30E-15 | 1.47E-14 | (loc_os02g10120.1 : 612.0) no description available & (q8gsm2\|lox23_horvu : 595.0) Lipoxygenase 2.3, chloroplast precursor (EC 1.13.11.12) (LOX2:Hv:3) - Hordeum vulgare (Barley) & (at3g45140 : 593.0) Chloroplast lipoxygenase required for wound-induced jasmonic acid accumulation in Arabidopsis.Mutants are resistant to Staphylococcus aureus and accumulate salicylic acid upon infection.; lipoxygenase 2 (LOX2); FUNCTIONS IN: lipoxygenase activity; INVOLVED IN: in 7 processes |
|  |  | *cl847.contig3_all* | -2.172978 | 1.40E-203 | 7.50E-202 | (at3g45140 : 953.0) Chloroplast lipoxygenase required for wound-induced jasmonic acid accumulation in Arabidopsis.Mutants are resistant to Staphylococcus aureus and accumulate salicylic acid upon infection.; lipoxygenase 2 (LOX2); FUNCTIONS IN: lipoxygenase activity; INVOLVED IN: in 7 processes |
|  |  | *cl847.contig6_all* | 1.8207239 | 2.30E-144 | 6.90E-143 | (loc_os02g10120.1 : 935.0) no description available & (at3g45140 : 929.0) Chloroplast lipoxygenase required for wound-induced jasmonic acid accumulation in Arabidopsis.Mutants are resistant to Staphylococcus aureus and accumulate salicylic acid upon infection.; lipoxygenase 2 (LOX2); FUNCTIONS IN: lipoxygenase activity; INVOLVED IN: in 7 processes |
|  |  | *cl907.contig2_all* | -0.291934 | 3.58E-48 | 3.99E-47 | (at1g55020 : 993.0) lipoxygenase, a defense gene conferring resistance Xanthomonas campestris; lipoxygenase 1 (LOX1) |
|  |  | *cl907.contig4_all* | 0.5697746 | 6.75E-29 | 4.90E-28 | (at1g55020 : 399.0) lipoxygenase, a defense gene conferring resistance Xanthomonas campestris; lipoxygenase 1 (LOX1) |
|  |  | *cl907.contig5_all* | 0.2934882 | 3.08E-12 | 1.18E-11 | (p37831\|lox1_soltu : 347.0) Lipoxygenase 1 (EC 1.13.11.12) - Solanum tuberosum (Potato) & (at1g55020 : 342.0) lipoxygenase, a defense gene conferring resistance Xanthomonas campestris; lipoxygenase 1 (LOX1) |
|  |  | *cl7820.contig1_all* | 0.1007745 | 1.40E-32 | 1.12E-31 | (q8gsm2\|lox23_horvu : 115.0) Lipoxygenase 2.3, chloroplast precursor (EC 1.13.11.12) (LOX2:Hv:3) - Hordeum vulgare (Barley) & (loc_os02g10120.1 : 113.0) no description available & (at3g45140 : 93.6) Chloroplast lipoxygenase required for wound-induced jasmonic acid accumulation in Arabidopsis.Mutants are resistant to Staphylococcus aureus and accumulate salicylic acid upon infection.; lipoxygenase 2 (LOX2); FUNCTIONS IN: lipoxygenase activity |
|  |  | *cl12121.contig1_all* | -0.633581 | 3.64E-15 | 1.82E-14 | (p37831\|lox1_soltu : 261.0) Lipoxygenase 1 (EC 1.13.11.12) - Solanum tuberosum (Potato) & (at3g22400 : 236.0) LOX5; FUNCTIONS IN: oxidoreductase activity, acting on single donors with incorporation of molecular oxygen, incorporation of two atoms of oxygen, lipoxygenase activity, iron ion binding, metal ion binding; INVOLVED IN: root development; LOCATED IN: chloroplast; EXPRESSED IN: 22 plant structures |
|  |  | *cl12121.contig2_all* | -1.061818 | 5.52E-19 | 2.89E-18 | (p37831\|lox1_soltu : 441.0) Lipoxygenase 1 (EC 1.13.11.12) - Solanum tuberosum (Potato) & (at3g22400 : 410.0) LOX5; FUNCTIONS IN: oxidoreductase activity, acting on single donors with incorporation of molecular oxygen, incorporation of two atoms of oxygen, lipoxygenase activity, iron ion binding, metal ion binding |
|  |  | *cl12121.contig3_all* | -1.237924 | 3.95E-42 | 3.93E-41 | (p37831\|lox1_soltu : 1111.0) Lipoxygenase 1 (EC 1.13.11.12) - Solanum tuberosum (Potato) & (at1g55020 : 1060.0) lipoxygenase, a defense gene conferring resistance Xanthomonas campestris; lipoxygenase 1 (LOX1) |
|  | *AOS* | *unigene5700_all* | -1.764302 | 2.28E-10 | 7.75E-10 | (at5g42650 : 632.0) Encodes a member of the cytochrome p450 CYP74 gene family that functions as an allene oxide synthase. This enzyme catalyzes dehydration of the hydroperoxide to an unstable allene oxide in the JA biosynthetic pathway. It shows a dual catalytic activity, the major one being a 13-AOS but also expressing a 9-AOS activity.; allene oxide synthase (AOS); FUNCTIONS IN: hydro-lyase activity, allene oxide synthase activity, oxygen binding |
|  |  | *unigene25377_all* | 0.7899037 | 1.42E-25 | 9.38E-25 | (at5g42650 : 475.0) Encodes a member of the cytochrome p450 CYP74 gene family that functions as an allene oxide synthase. This enzyme catalyzes dehydration of the hydroperoxide to an unstable allene oxide in the JA biosynthetic pathway. It shows a dual catalytic activity, the major one being a 13-AOS but also expressing a 9-AOS activity.; allene oxide synthase (AOS); FUNCTIONS IN: hydro-lyase activity, allene oxide synthase activity, oxygen binding |
|  | *AOC* | *cl16780.contig1_all* | -0.679341 | 8.15E-13 | 3.21E-12 | (at3g25780 : 220.0) Encodes allene oxide cyclase, one of the enzymes involved in jasmonic acid biosynthesis. One of four genes in Arabidopsis that encode this enzyme. mRNA expression is upregulated in senescing leaves |
|  |  | *cl16780.contig2_all* | 1.5703442 | 2.19E-05 | 4.95E-05 | (at3g25770 : 164.0) Encodes allene oxide cyclase. One of four genes in Arabidopsis that encode this enzyme, which catalyzes an essential step in jasmonic acid biosynthesis. Gene expression is induced during senescence, a process that involves jasmonic acid signalling pathway |
|  | *OPR* | *unigene3623_all* | -0.49414 | 4.07E-15 | 1.81E-14 | (loc_os08g35740.1 : 540.0) no description available & (at2g06050 : 509.0) Encodes a 12-oxophytodienoate reductase that is required for jasmonate biosynthesis. Mutants are male sterile and defective in pollen dehiscence. Shows activity towards 2,4,6-trinitrotoluene.; oxophytodienoate-reductase 3 (OPR3); FUNCTIONS IN: 12-oxophytodienoate reductase activity |
|  |  | *unigene8154_all* | 0.690262 | 2.72E-12 | 1.17E-11 | (at1g76680 : 104.0) Encodes a member of an alpha/beta barrel fold family of FMN-containing oxidoreductases. One of the closely related 12-oxophytodienoic acid reductases. Up-regulated by senescence and jasmonic acid. Predicted to be a cytosolic protein.; 12-oxophytodienoate reductase 1 (OPR1); FUNCTIONS IN: 12-oxophytodienoate reductase activity |
|  |  | *unigene12250_all* | -1.005236 | 3.14E-22 | 1.86E-21 | (at1g76680 : 152.0) Encodes a member of an alpha/beta barrel fold family of FMN-containing oxidoreductases. One of the closely related 12-oxophytodienoic acid reductases. Up-regulated by senescence and jasmonic acid. Predicted to be a cytosolic protein.; 12-oxophytodienoate reductase 1 (OPR1); FUNCTIONS IN: 12-oxophytodienoate reductase activity |
|  |  | *unigene21534_all* | 0.6264432 | 1.24E-11 | 4.57E-11 | (at1g76690 : 105.0) Encodes one of the closely related 12-oxophytodienoic acid reductases. This enzyme is not expected to participate in jasmonic acid biosynthesis because during in vitro assays, it shows very little activity with the naturally occurring OPDA isomer. Shows activity towards 2,4,6-trinitrotoluene. Expressed predominately in root. Predicted to be a cytosolic protein.; 12-oxophytodienoate reductase 2 (OPR2) |
|  |  | *unigene26083_all* | 2.842572 | 2.52E-13 | 1.02E-12 | (at1g76690 : 533.0) Encodes one of the closely related 12-oxophytodienoic acid reductases. This enzyme is not expected to participate in jasmonic acid biosynthesis because during in vitro assays, it shows very little activity with the naturally occurring OPDA isomer. Shows activity towards 2,4,6-trinitrotoluene. Expressed predominately in root. Predicted to be a cytosolic protein.; 12-oxophytodienoate reductase 2 (OPR2) |
|  |  | *cl7812.contig2_all* | -0.78705 | 1.41E-07 | 3.90E-07 | (loc_os06g11210.1 : 97.4) no description available & (at1g76680 : 87.8) Encodes a member of an alpha/beta barrel fold family of FMN-containing oxidoreductases. One of the closely related 12-oxophytodienoic acid reductases. Up-regulated by senescence and jasmonic acid. Predicted to be a cytosolic protein.; 12-oxophytodienoate reductase 1 (OPR1); FUNCTIONS IN: 12-oxophytodienoate reductase activity |
|  |  | *cl7812.contig3_all* | 0.4200108 | 2.00E-42 | 2.29E-41 | (loc_os06g11210.1 : 97.4) no description available & (at1g76680 : 87.8) Encodes a member of an alpha/beta barrel fold family of FMN-containing oxidoreductases. One of the closely related 12-oxophytodienoic acid reductases. Up-regulated by senescence and jasmonic acid. Predicted to be a cytosolic protein.; 12-oxophytodienoate reductase 1 (OPR1); FUNCTIONS IN: 12-oxophytodienoate reductase activity |
|  |  | *cl10591.contig1_all* | 2.7169013 | 1.32E-11 | 4.83E-11 | (at1g76680 : 491.0) Encodes a member of an alpha/beta barrel fold family of FMN-containing oxidoreductases. One of the closely related 12-oxophytodienoic acid reductases. Up-regulated by senescence and jasmonic acid. Predicted to be a cytosolic protein.; 12-oxophytodienoate reductase 1 (OPR1); FUNCTIONS IN: 12-oxophytodienoate reductase activity |
|  |  | *cl10591.contig2_all* | -0.399787 | 1.89E-13 | 7.73E-13 | (at1g09400 : 241.0) FMN-linked oxidoreductases superfamily protein; FUNCTIONS IN: oxidoreductase activity, FMN binding, catalytic activity |
|  |  | *cl11449.contig2_all* | -0.779365 | 1.13E-17 | 5.62E-17 | (loc_os06g11240.1 : 116.0) no description available & (at1g76690 : 113.0) Encodes one of the closely related 12-oxophytodienoic acid reductases. This enzyme is not expected to participate in jasmonic acid biosynthesis because during in vitro assays, it shows very little activity with the naturally occurring OPDA isomer. Shows activity towards 2,4,6-trinitrotoluene. Expressed predominately in root. Predicted to be a cytosolic protein.; 12-oxophytodienoate reductase 2 (OPR2) |
