# Supplementary material for: Expression Analysis of TCP Transcription Factor Family in Autopolyploids of Chrysanthemum nankingense
Source: Front Plant Sci. 2022 Jun 2;13:860956. doi: 10.3389/fpls.2022.860956 (PMC9201386; doi:10.3389/fpls.2022.860956)
Supplement: Supplementary file 2 [file Data_Sheet_2.docx]

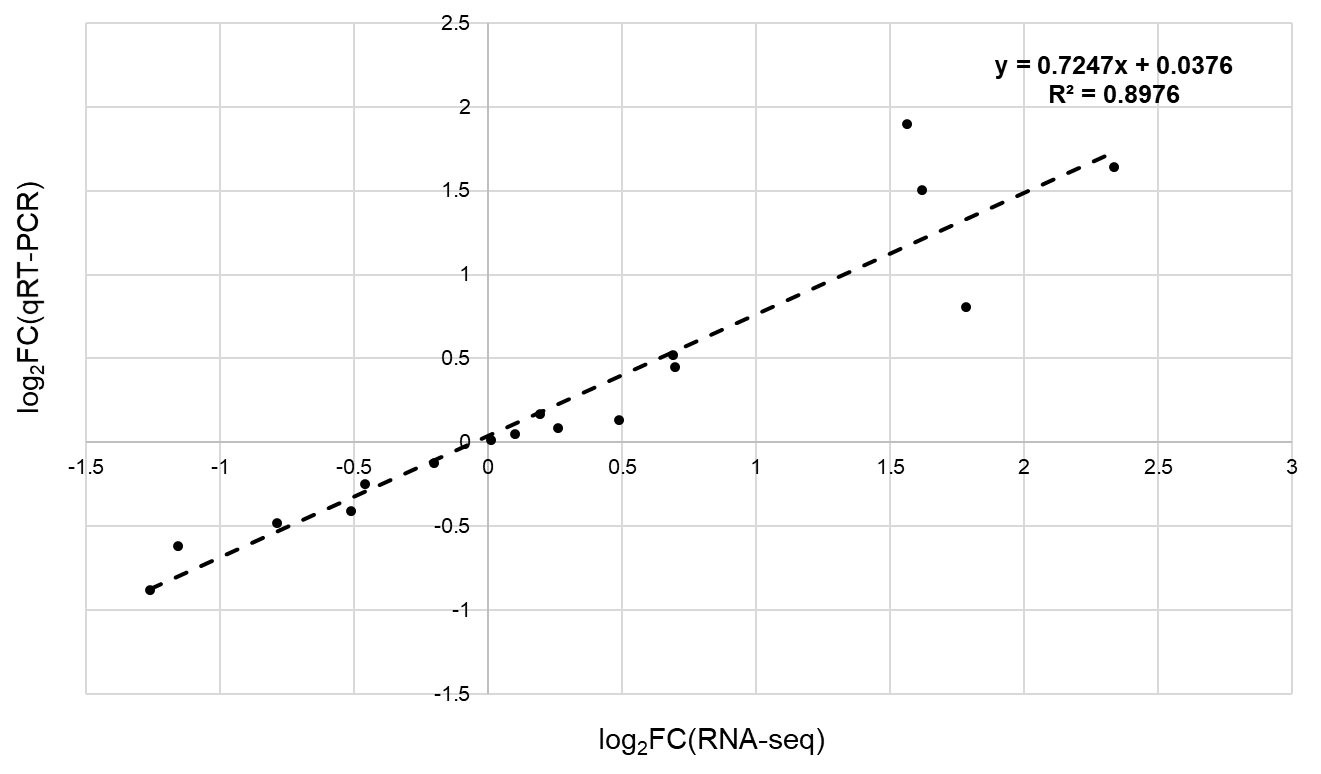


Figure S1. Correlation analysis of qRT-PCR and RNA-seq.

Using the log2^FC^ values of RNA-seq of all *TCP* genes as the x-axis and the log2^FC^ values of qRT-PCR as the y-axis. The linear regression equation y = 0.7247x+0.0376, R^2^ = 0.8976 was obtained by fitting.
